# Telling ecological networks apart by their structure: An environment-dependent approach

**DOI:** 10.1371/journal.pcbi.1007787

**Published:** 2020-04-23

**Authors:** Chuliang Song, Serguei Saavedra

**Affiliations:** Department of Civil and Environmental Engineering, MIT, Cambridge, Massachusetts, United States of America; University of Illinois at Urbana-Champaign, UNITED STATES

## Abstract

The network architecture of an ecological community describes the structure of species interactions established in a given place and time. It has been suggested that this architecture presents unique features for each type of ecological interaction: e.g., nested and modular architectures would correspond to mutualistic and antagonistic interactions, respectively. Recently, Michalska-Smith and Allesina (2019) proposed a computational challenge to test whether it is indeed possible to differentiate ecological interactions based on network architecture. Contrary to the expectation, they found that this differentiation is practically impossible, moving the question to why it is not possible to differentiate ecological interactions based on their network architecture alone. Here, we show that this differentiation becomes possible by adding the local environmental information where the networks were sampled. We show that this can be explained by the fact that environmental conditions are a confounder of ecological interactions and network architecture. That is, the lack of association between network architecture and type of ecological interactions changes by conditioning on the local environmental conditions. Additionally, we find that environmental conditions are linked to the stability of ecological networks, but the direction of this effect depends on the type of interaction network. This suggests that the association between ecological interactions and network architectures exists, but cannot be fully understood without attention to the environmental conditions acting upon them.

## Author summary

It has been suggested that different types of species interactions lead to ecological networks with different architectures. For example, mutualistic and antagonistic interaction networks have been shown to have nested and modular architectures, respectively. Importantly, this differentiation can provide clues about the link between the dynamics and structures shaping ecological communities. Recently, Michalska-Smith and Allesina (2019) turned this assumption into a serious computational challenge for the scientific community. Here, we embrace this challenge. We confirm that network architecture alone is not enough to differentiate interaction networks. However, we show that network architectures can differentiate between mutualistic and antagonistic interaction networks by using information about their local environmental conditions. In other words, ignoring environmental information throws out the predictable patterns of network architectures along environmental gradients. Thus, this response is also a reminder that ecological networks may only make sense in the light of environmental information.

## Introduction

A local ecological community is the ensemble formed by the co-occurring and interacting species in a given location and time [1]. Each of these ecological communities forms a network with an architecture defined by the species (nodes) and their interactions (links) [2]. Over the last decades, ecologists have intensively documented the architecture of antagonistic interaction networks (e.g., host-parasite and plant-herbivore interaction networks) and mutualistic interaction networks (e.g., plant-ant, plant-pollinator, and plant-dispersal interaction networks) [3, 4]. It has been shown that the network architecture of antagonistic and mutualistic interactions tend to be more modular and nested than expected by chance alone, respectively [3, 5–7]. However, it has also been shown that this potential generality can be easily broken [8–10]. These contrasting ideas have raised the question of whether interaction networks can, in fact, be differentiated based on their network architectures. If true, these architectures can be used to better understand the patterns shaping the biodiversity that we observe in nature [10, 11].

To answer the questions above, a recent study has proposed a computational perspective [12]. Specifically, this research has posed a relatively simple question: do the network architectures of antagonistic and mutualistic interactions differ consistently and detectably? Before we introduce their answer, let us walk through some background of what answers are typically expected. It is well established that antagonistic and mutualistic interactions generate qualitatively different dynamical behavior [13–18]. Therefore, if the network architectures ought to reflect the underlying ecological dynamics, we should anticipate important architectural differences between antagonistic and mutualistic interactions. These differences have been empirically found even among different classes of non-ecological networks [19], and theoretically predicted between antagonistic and mutualistic interactions [3]. Yet, despite the wide-held expectation about these differences in ecology, the few general comparison studies have suffered either from ill-defined methodologies or small data sets [12, 20].

In contrast to the expectation above, after trying out many sophisticated computational methods, Michalska-Smith and Allesina [12] could not find a systematic difference in the binary network architecture of antagonistic and mutualistic interactions. These new results have shown that the idea that mutualistic interactions are nested and antagonistic interactions are modular is an oversimplifying representation of nature [21]. However, a question remains: why there was no difference? One possibility, of course, is that simply there is no systematic difference whatsoever. Another explanation is that we need a more powerful algorithm to tell these interaction networks apart. However, given that these algorithms can easily tell apart different classes of non-ecological networks [12, 19], it is unlikely that this is the right explanation. A third explanation is that there is a key missing factor that systematically affects the network architecture and interactions of ecological communities, which needs to be taken into account in order to differentiate such architectures.

Ecological research has always stipulated that the occurrence of species does not only depend on the biotic factors described in ecological communities, but also depends on the abiotic factors established by the environment [22, 23]. This explanation is rooted upon the general idea of environmental filtering—the environment is a major force shaping almost all ecological systems [1, 24, 25]. For example, temperature variability affects both species interactions [26, 27] and network structures [21, 28, 29]. Thus, under this rationale, the network architecture itself may not carry enough information about the interaction types if the environment is unknown [30]. That is, a locked box (network architecture) is useless unless one also has the right key (environmental conditions). In statistics, this issue is known as the omitted-variable bias [31]. Note that because non-ecological networks may not depend as strongly on the environment as ecological networks do, this explanation may also reveal why non-ecological networks can be easily told apart. In this direction, previous work has already shown that incorporating environmental conditions (such as temperature variability) can explain the diversity of architectures found in mutualistic communities [32]. Therefore, we speculate that the reason why Michalska-Smith and Allesina [12] failed to differentiate the network architecture of mutualistic and antagonistic interactions is because they did not consider environmental conditions. Note that this explanation does not invalidate the fact that ecological interactions cannot be differentiated based on their architectures alone as Michalska-Smith and Allesina [12] have already shown.

To test whether adding environmental information can help us to differentiate the architecture of ecological interaction networks, we analyze a world-wide collection of antagonistic and mutualistic communities together with their local environmental conditions. Following the methodology proposed by Michalska-Smith and Allesina [12], we show that antagonistic and mutualistic interactions can, in fact, be told apart by adding environmental information. We then show that environmental information alone (i.e., removing the observed network architecture by randomizing interactions) cannot differentiate between antagonistic and mutualistic interactions, confirming that both network architecture and environmental conditions are essential components of ecological communities. Finally, we show the relationship between environmental information and the stability properties of ecological networks, providing an explanation of why environmental conditions can be a key confounding factor.

## Methods

### Empirical datasets and choice of metrics

The original data set used by Michalska-Smith and Allesina [12] did not include the location where each of the ecological networks was sampled. Hence, we based our analysis on the data set of ecological networks found in the public repository web-of-life.es, which has this type of information. We then extracted the environmental data of each location from the public repository WorldClim [33] using the geographical information provided in web-of-life.es. Using these repositories, we compiled 177 mutualistic interaction networks and 75 antagonistic interaction networks together with their environmental data (see S1 Supporting Information for details).

To describe the network architecture of our compiled data set, we used the same network metrics as in Michalska-Smith and Allesina [12]: the largest eigenvalue (λ_1_) of the binary interaction matrix as an indicator of nestedness [34], and the second largest eigenvalue (λ_2_) of the binary interaction matrix as an indicator of modularity [35]. The details of the computations can be found in Michalska-Smith and Allesina [12] or in S1 Supporting Information. Additionally, because network size and connectance are biased by how networks are sampled, Michalska-Smith and Allesina [12] computed null expectations in order to control for the bias. Specifically, Michalska-Smith and Allesina [12] have used two null models: *Erdős-Rényi* randomization (denoted as ^er^), where network size and connectance are preserved; and the *Configuration* randomization (denoted as ^cm^), where network size, connectance, and degree distribution are preserved. Thus, following Michalska-Smith and Allesina [12], we used three network metrics: the relative error of the largest eigenvalue given the configuration randomization ($1-\lambda_{1}^{\text{cm}}/\lambda_{1}$), the relative error of the largest eigenvalue given the Erdős-Rényi randomization ($1-\lambda_{1}^{\text{er}}/\lambda_{1}$), and the relative error of the second largest eigenvalue given the Erdős-Rényi randomization ($1-\lambda_{2}^{\text{er}}/\lambda_{2}$).

**Fig 1:**
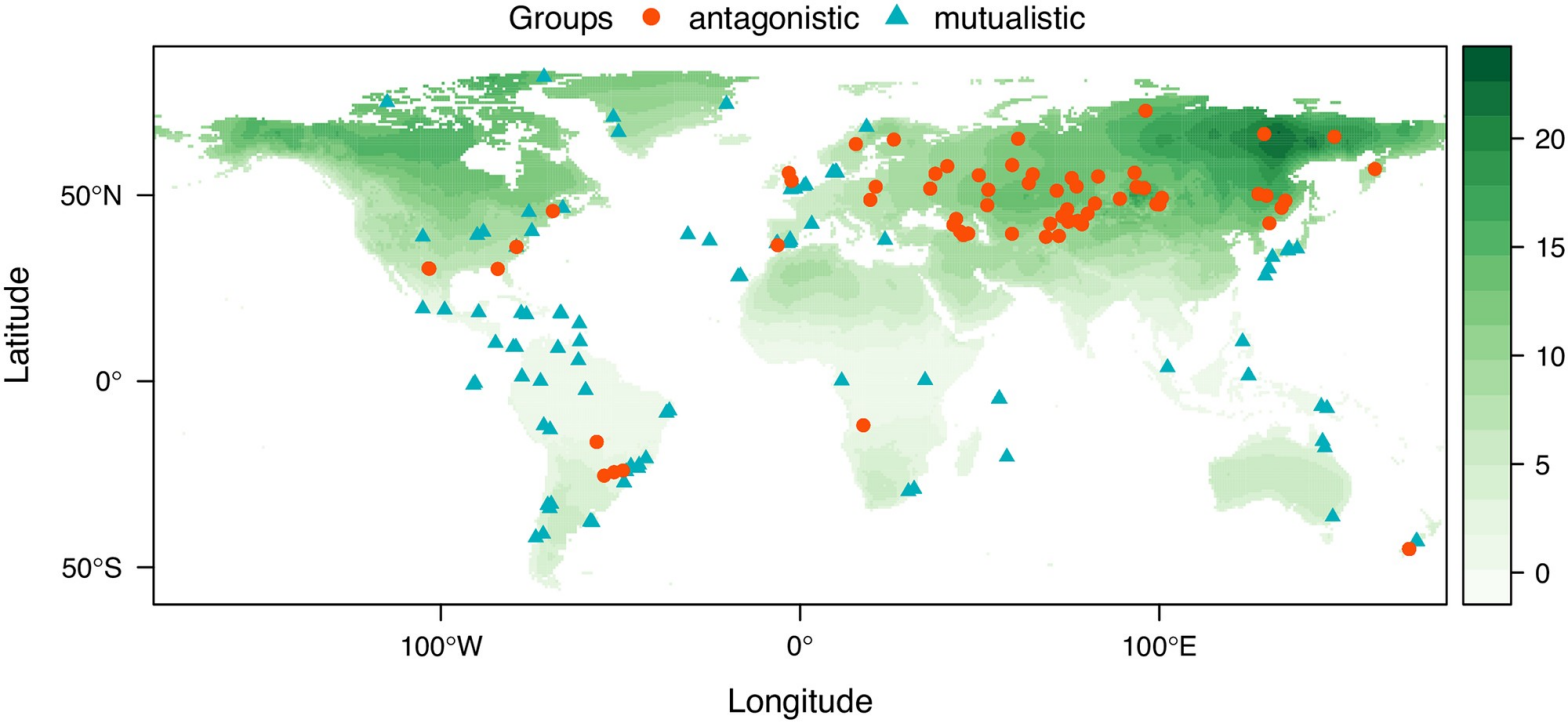
The location and local temperature variability of each of the ecological networks in our data set. The figure shows the location of antagonistic interaction networks (red circles) and mutualistic interaction networks (blue triangles) extracted from the web-of-life.es. The x-axis and y-axis represent the longitude and latitude, respectively. The color bar represents the temperature variability measured as the standard deviation of the yearly temperature in Celsius at a given location (taken from WorldClim). Green and orange colors correspond to higher and lower temperature variability, respectively. This map was plotted with raster package [76]. This map was published under a CC-BY license. The data are provided as Supplementary Material.

To take into account the environmental conditions of each ecological network of our compiled data set, we needed to choose an indicator of the environmental conditions. While WorldClim provides 19 environmental variables [33], many of these variables are strongly correlated (S1 Supporting Information). For example, temperature variability has a correlation stronger than 0.7 with 11 out of 19 environmental variables. This introduces the statistical issue known as multicollinearity [36]. Although some sophisticated statistical methods can deal with multicollinearity [37, 38], they work only under very limited scenarios and are not directly interpretable [39]. Thus, to simplify the analysis, we used temperature variability—defined as the standard variation of the annual temperature fluctuation (units: Celsius). Fig 1 shows the geographical distribution of these ecological networks and their local temperature variability. This choice is based on the strong empirical evidence that temperature variability is an important indicator of environmental stress and has profound impacts on both species interactions and ecological communities. [32, 40–43]. For example, focusing on the interaction level, the Stress Gradient Hypothesis states that species interactions (e.g. mutualistic or competitive) may switch to a different type under different temperatures [26, 27, 44, 45]. Focusing on the community level, it has been shown that temperature variability affects almost all aspects of the community, such as productivity [46, 47], phenology [42, 48], and network architecture [20, 32]. Furthermore, to validate our results, we repeated our main analysis using other environmental variables in S1 Supporting Information.

### Differentiating networks

To differentiate ecological interaction networks, we followed again Michalska-Smith and Allesina [12] and used Principal Component Analysis (PCA) to map the multiple network metrics and temperature variability into the plane defined by the first two principal components [36]. PCA facilitates visualizing how similar the metrics of the ecological networks are by looking at the distances between the mapped networks on the plane. Although PCA is a linear method [36], it has already been shown to perform well in telling apart non-ecological networks [12]. Note that all variables are scaled to zero mean and unit variance in the PCA (see details in S1 Supporting Information). We have also replicated our analysis using the t-distributed stochastic neighbor embedding (t-SNE), a nonlinear dimensionality reduction method (S1 Supporting Information).

**Fig 2:**
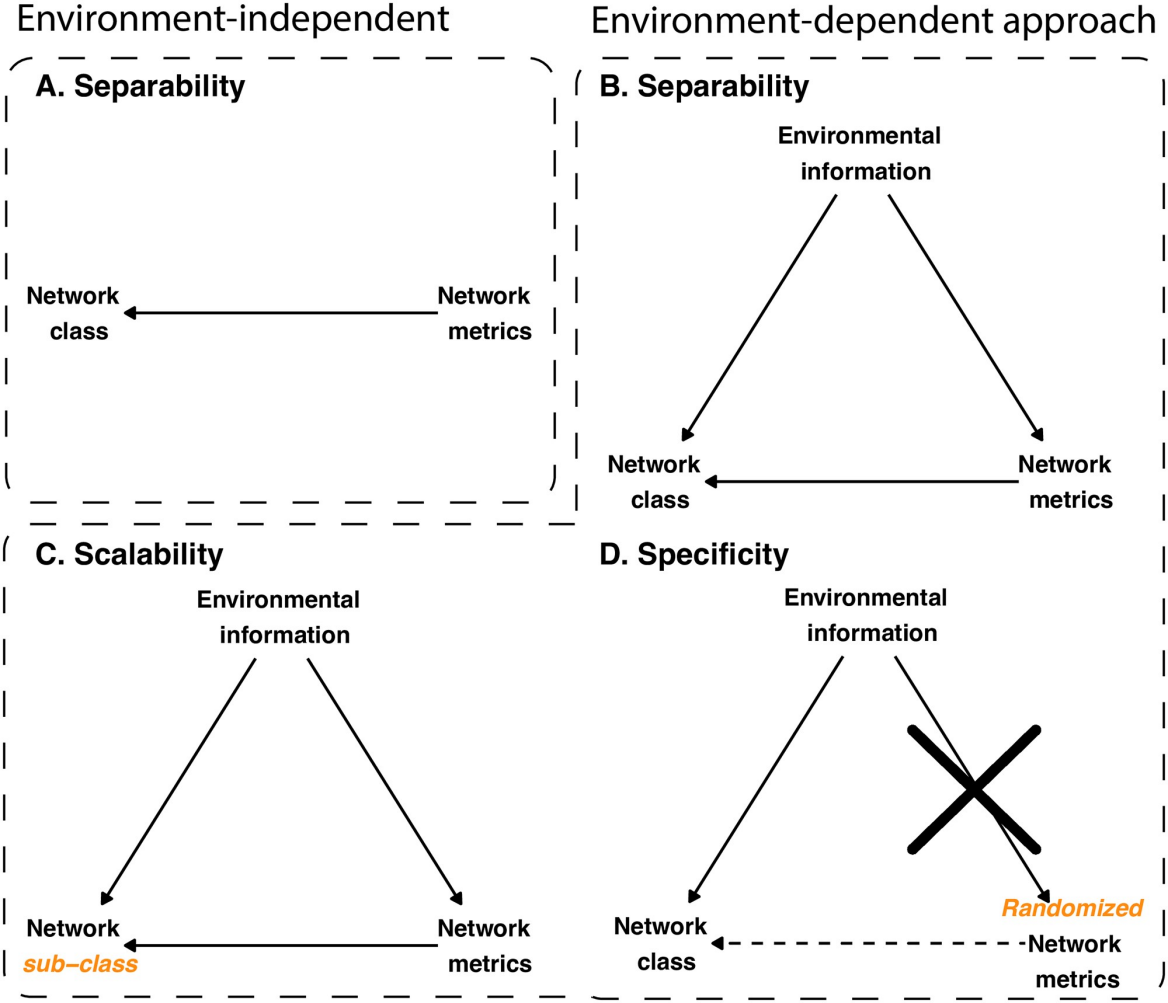
Illustration of the environment-dependent approach. This figure illustrates the structural graphs that the environment-independent and -dependent approaches use to test the three differentiation criteria. Panels (**A**-**B**) focus on the separability criterion. Panel (**A**) shows that in the environment-independent approach, Network Metrics alone are used to differentiate Network Class. Instead, Panel (**B**) shows that in the environment-dependent approach, both Network Metrics and Environmental Information are used to differentiate Network Class. Note that Environmental Information becomes a confounding factor of Network Metrics and Network Class. Panel (**C**) focuses on the scalability criterion in the environment-dependent approach. Specifically, whether Network Metrics and Environmental Information can also differentiate Network Class into sub-classes. Panel **D** focuses on the specificity criterion in the environment-dependent approach. The Network Metrics are generated from randomized network architectures, which removes the effect of Environmental Information on Network Metrics. This randomization also weakens the effect of Network Metrics on Network Class. Note that Network Class becomes a collider between Environmental Information and Network Metrics (see S1 Supporting Information for further details). That is, Environmental Information and Network Metrics are potentially dependent conditional on Network Class [49].

It is also important to mention that in order to move to an environment-dependent approach, it is necessary to depart from the structural model introduced by Michalska-Smith and Allesina [12]. Specifically, Michalska-Smith and Allesina [12] studied the capacity to differentiate ecological interactions based on network metrics alone (Fig 2A). This structural model only has one *chain*: Metrics → Interactions [49]. In comparison, the environment-dependent approach takes network metrics and environmental information together (Fig 2B). This is built on the rationale that environmental conditions impact both the type of interactions [26, 27, 45, 50] and the network architecture [32, 51, 52] of ecological interaction networks. Hence, this new structural model has two chains (Metrics → Interactions and Metrics → Temperature → Interactions) and one *collider* (Metrics → Interactions ← Temperature). As we explain below, this new model carries important statistical constraints that need to be taken into account when testing cause-effect relationships.

We used the three criteria introduced by Michalska-Smith and Allesina [12] to test whether an environment-dependent approach can be used to differentiate ecological interactions. The first criterion is *separability*: whether the metrics can separate antagonistic and mutualistic interaction networks. We tested for separability in our data set using both environment-independent and -dependent approaches (Fig 2A and 2B). Note that we termed this criterion “separability” instead of “generality” as used in Michalska-Smith and Allesina [12]. This is because we only focus on ecological networks here, instead of many other network types as in Michalska-Smith and Allesina [12]. The second criterion is *scalability*: whether these metrics can differentiate ecological networks across hierarchical levels [53]. For example, whether a plant-pollinator network (a sub-class of mutualistic interaction networks) are closer to other plant-pollinator networks in the PCA than to other sub-classes of mutualistic interaction networks (e.g., plant-ant, seed-dispersal) (see Fig 2C for the illustration). The third criterion is *specificity*: whether the randomized network architectures cannot be differentiated in the PCA. This is rooted on the rationale that a randomized network architecture should not be informative of underlying ecological processes.

In the first two criteria, the environment-dependent approach of this study and the approach of Michalska-Smith and Allesina [12] differ only from the perspective of adding or not a conditional variable (namely Temperature). However, the third criterion imposes additional constraints not shared between approaches. That is, following the environment-dependent approach, Fig 2D illustrates that randomizing the networks would remove the effect that the environment has on network metrics, while weakening the effect that network metrics has on ecological interactions. Yet, if we condition on the collider Interactions, then we will establish a spurious association between Temperature and Metrics [49]. Note that Michalska-Smith and Allesina [12] proposed to use the scaling of the empirical data for the PCA of the randomized data. While this procedure works as a clever classifier, unfortunately, it cannot be applied to the environment-dependent approach as it carries the expectation of all variables, acting effectively as a conditional on the collider (S1 Supporting Information). Importantly, this third criterion (using the scaling of the randomized data) addresses the geographical sampling bias in our data set: most antagonistic networks are sampled from regions with higher temperature variability than those from mutualistic networks (Fig 1 and S1 Supporting Information). Specifically, if the differentiation of ecological networks is an artifact of adding environmental conditions, then the same analysis should still differentiate the randomized networks when the environmental condition is fixed, which would violate the specificity criterion. To further validate the test on specificity, we have also run additional analysis on specificity where the environmental conditions are randomized (S1 Supporting Information).

### Inferring the effects of temperature variability

The PCA can reveal *how* to differentiate ecological communities, however, it tells us little about *why* the networks can be differentiated. Therefore, to analyze the effects of temperature variability on network architecture, we investigated the relationship between temperature variability and three standard stability metrics of ecological networks: the largest eigenvalue (λ_1_), the second largest eigenvalue (λ_2_), and the relative size of the feasibility domain of the intra-guild competition (Ω). The first two metrics—λ_1_ and λ_2_—are related to dynamical stability [54]. The larger these eigenvalues are, the more dynamically stable are these networks. The third metric (Ω) is related to the structural stability of intra-guild competition [55, 56]. These metrics measure the overall resource competition among competitive agents [42, 57, 58]. The stronger the competition among competitive agents, the smaller the structural stability of feasibility of the community [58, 59]. S1 Supporting Information provides details on how to compute these measures. Note that all these metrics can be estimated with the binary information contained in our data set.

To estimate the effects of temperature variability on these three stability metrics, we performed a multiple regression analysis, where community size and connectance are used as independent variables. We performed linear regression in order to be able to compare our results with the PCA, which extracts mostly the linear relationships among variables [36]. We additionally validated the estimation of effects by changing the independent variables (S1 Supporting Information). Finally, we compared the effects of temperature variability using the randomized network metrics (S1 Supporting Information).

## Results

**Fig 3:**
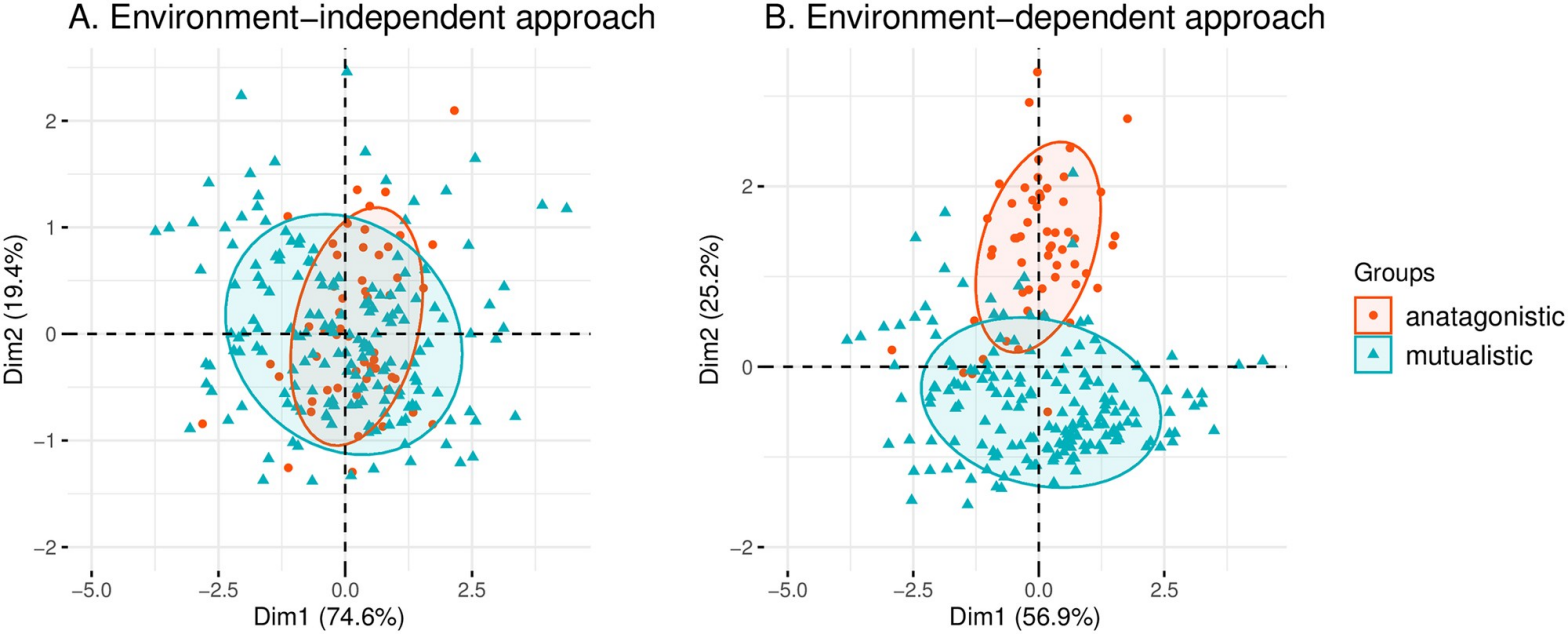
Differentiating ecological interaction networks. This figure examines the separability of network interactions under environment-independent and -dependent approaches. Using a Principal Component Analysis, mutualistic (blue triangles) and antagonistic (red circles) interaction networks are mapped into the first two principal components given by the chosen metrics. Each ellipse contains approximately 68% of the networks in each class. Panel (**A**) shows that antagonistic and mutualistic interaction networks cannot be differentiated when only network metrics are used (environment-independent approach). Instead, Panel (**B**) shows that these interaction networks can be differentiated when both network metrics and environmental information are used (environment-dependent approach).

We found that it is indeed possible to differentiate ecological interactions by combining network metrics and environmental information. Focusing on separability, we confirmed that the network metrics alone cannot tell apart antagonistic and mutualistic interaction networks. Fig 3A shows that these two classes of interaction networks overlap strongly on the PCA map. However, once we add the environmental information of temperature variability, the antagonistic and mutualistic interaction networks are clearly separated (Fig 3B).

**Fig 4:**
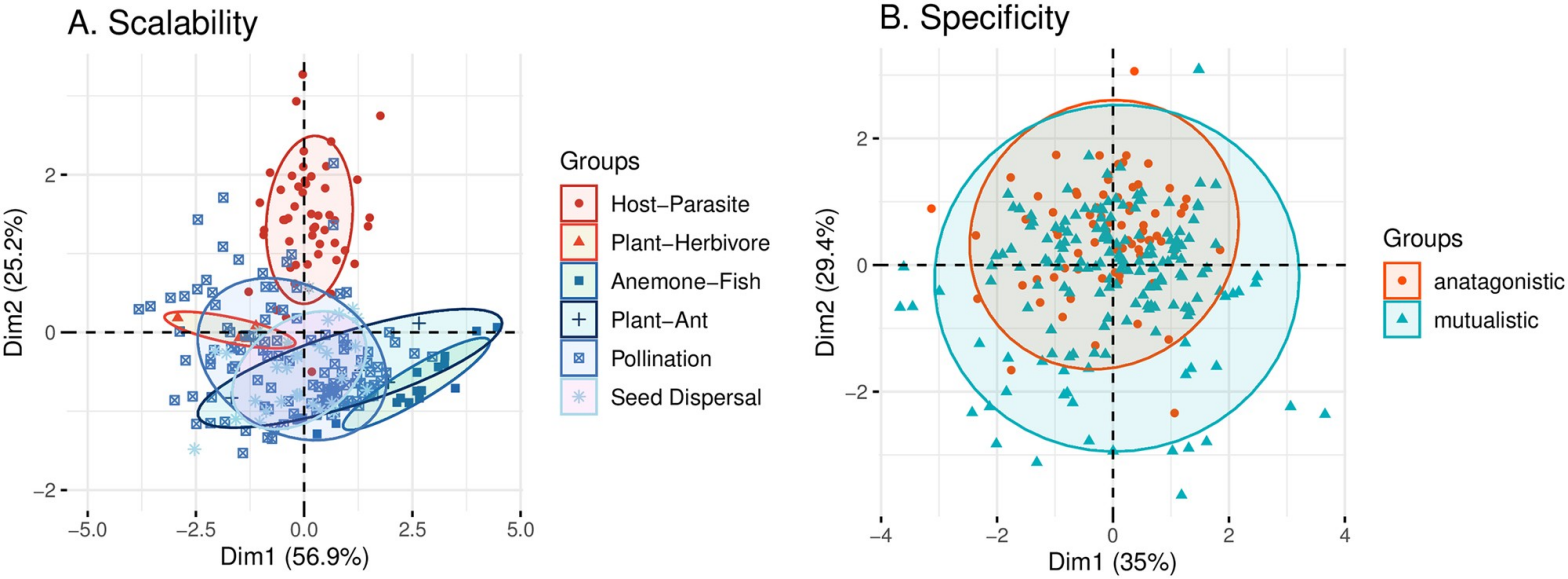
Testing the scalability and specificity of the environment-dependent approach. Panel (**A**) shows that the environment-dependent approach is scalable. The figure corresponds to the PCA map (first two principal components) using the empirical data following the environment-dependent approach. Antagonistic class: red circles and red triangles represent host-parasite and plant-herbivore sub-classes, respectively. Mutualistic class: blue squares, blue crosses, blue crossed squares, and blue stars correspond to anemone-fish, plant-ant, plant-pollinator, and plant-dispersal sub-classes. The PCA shows that the network sub-classes can be differentiated and they are closer to other network sub-classes within their own interaction class. Panel (**B**) shows that the environment-dependent approach follows specificity. This panel shows the PCA map (first two principal components) derived from the randomized network metrics and following the environment-dependent approach. Each empirical network is randomized using the Erdős-Rényi model. As expected, the randomized networks cannot be differentiated even with environmental information. Importantly, this panel confirms that the geographical sampling bias of ecological networks does not strongly influence our results. Note that only one randomization per network is shown here for simplicity; a systematic test of specificity can be found in S1 Supporting Information. Also note that different from the specificity criterion used by Michalska-Smith and Allesina [12], the PCA map is scaled according to the randomized data. Otherwise, because of the nature of the structural model (Fig 2), scaling using the empirical data would establish a spurious association between temperature and network metrics (S1 Supporting Information).

Then we test the scalability and specificity of the environment-dependent approach. Fig 4A reveals that interaction networks from a sub-class are closer to other network sub-classes from the same interaction class than from the other interaction class, confirming the scalability of the approach. Instead, Fig 4B shows that the randomized network architectures strongly overlap on the PCA map, confirming the specificity of the approach. Note that for simplicity, we have only shown one realization of randomization for each network in Fig 4B; a systematic test of the specificity can be found in S1 Supporting Information. Overall, these results show that an environment-dependent approach can allow us to statistically differentiate ecological interaction networks using network architectures.

**Fig 5:**
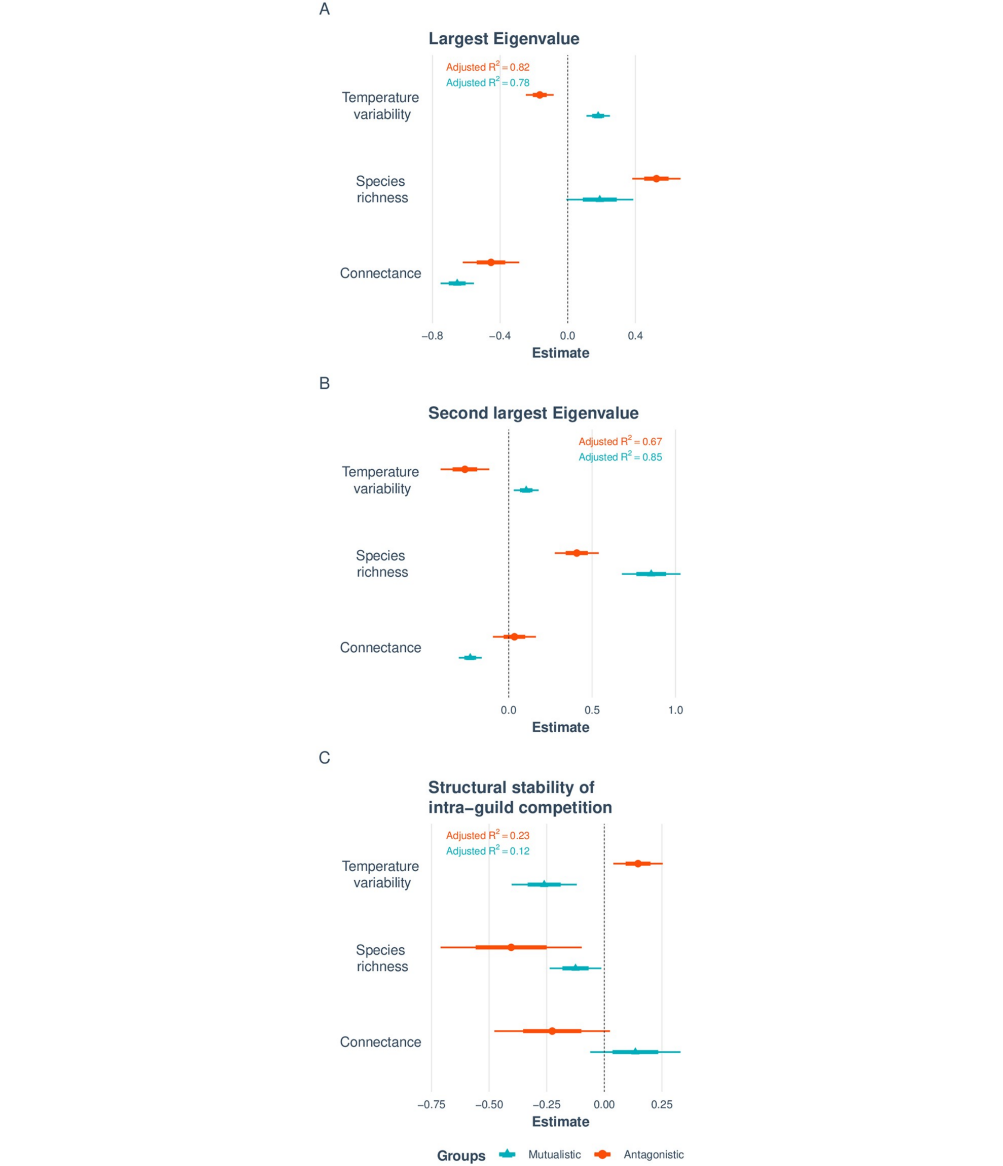
Effect of temperature variability on network stability. Panels (**A-C**) summarize the results of regressing temperature variability on three different metrics of network stability: the largest eigenvalue, the second largest eigenvalue, and the structural stability of feasibility. Mutualistic and antagonistic networks are shown in red circles and in blue triangles, respectively. The adjusted *R*^2^ for each regression model is shown on the top. All variables are scaled. The thick interval indicates 1 standard deviation, whereas the thin interval indicates 2 standard deviations. For all metrics of network stability, increasing temperature variability significantly decreases (increases) network stability for mutualistic (antagonistic) communities. Details can be found in S1 Supporting Information.

Moving to potential explanations about the association of environmental conditions with network architecture and type of interaction network (Fig 2B), we found that temperature variability has clear opposite effects on the stability of mutualistic and antagonistic networks. Specifically, Fig 5 (see also S1 Supporting Information) shows that increasing temperature variability increases the dynamical stability of mutualistic networks but decreases their structural stability of feasibility. However, this effect is completely in the opposite direction in antagonistic networks. These opposite effects are robust to different regression models (S1 Supporting Information); and, as expected, disappear in randomized networks (S1 Supporting Information).

## Discussion

Michalska-Smith and Allesina [12] have cleverly framed the problem of differentiating ecological interactions by their network architecture under the metaphor “can one hear the shape of a drum” [60]. That is, whether the sounds from a drum contain enough information to infer the shape of the drum. But because the sounds change in different media, we cannot infer the shape of a drum without knowing the surrounding media (a.k.a. environment). The same logic applies to ecological networks, as they are not merely the product of internal dynamical processes but also of the external environmental conditions [1, 4]. In other words, the architecture of a network is much less useful without knowing the environmental pressures acting upon a community [30]. Following this rationale, we have confirmed that antagonistic and mutualistic communities cannot be differentiated using network metrics alone, but this differentiation becomes possible by adding environmental information.

Our findings are consistent with the literature. For example, it has been theoretically predicted that all else being equal, the more nested a mutualistic interaction network, the lower its dynamical stability and the larger its structural stability of feasibility [61–63]. On top of this, it has been empirically shown that more nested mutualistic interaction networks are typically located in more variable environments [32]. Hence, it can be naturally expected that temperature variability is associated with the architecture of ecological networks.

It is worth noting that the heart of the statistical issue presented here is known as the Simpson’s paradox [36]. In brief, the Simpson’s paradox states that a given association (positive, negative, or null) between two separate variables may change when conditioning on a third variable [64]. It has been shown that network architecture and type of interaction network have no association; however, when one conditions on environmental variability, this association becomes significant. In other words, ignoring temperature variability is throwing out the opposing and predictable patterns of network architectures along environmental gradients [21, 32]. Thus, we strongly believe that ecological networks should be analyzed under an environment-dependent approach [30].

An important limitation of our statistical analysis is that we have only proved the association but not the causation. That is, environment is only one out of many confounding factors that affect both species interactions and network architectures. Thus, we cannot rule out the possibility that the patterns between temperature variability and network architectures are caused by some other external factor. For example, another possible interpretation of the statistical results reported here is that network architectures vary along the latitude, forming a U-shaped curve for both antagonistic and mutualistic networks. This interpretation can be seen as an extension of the Latitudinal Diversity Gradient to network architectures [65, 66]. Similarly, the level of human impact may also be an important factor [67, 68]. This implies that future studies should index these potential confounding factors so that ecological networks can be analyzed under a more general context-dependent approach, which may eventually lead to a deeper causal understanding. Another important limitation of our statistical analysis is the inherent geographical sampling bias of ecological networks (Fig 1 and S1 Supporting Information). We have found strong signals that ecological networks change along the environmental gradient despite the sampling bias (Figs 3–5). Additionally, we confirmed that while this sampling bias increases our ability to tell apart networks, the extent of the increment is significantly less compared to the case when the network architectures and the environmental information are combined (S1 Supporting Information). Nonetheless, it would be ideal if future collections of empirical networks would address this sampling issue.

Beyond the technical issue of statistics, solving the computational challenge of differentiating ecological communities based on network architectures can also shed new light onto the generating processes of ecological networks. Several studies have proposed many theoretical hypotheses to explain the architecture of ecological networks, ranging from ecological dynamics, evolutionary processes, to statistical artefacts [3, 10, 61, 62, 69–73]. The diversity of such theoretical explanations makes it hard to point out whether the architectural properties of ecological networks do reflect the underlying ecological dynamics of the communities they represent [10, 11, 72, 74, 75]. However, the computational challenge raised by Michalska-Smith and Allesina [12] provides a unique opportunity to rigorously test which factors are strongly associated with network architectures. Our results suggest that both the internal dynamics and the environmental conditions contribute to the generating process of ecological networks. Of course, these concepts are not new [22, 23], yet these computation-based insights serve as a central reminder that general conclusions on ecological networks cannot be derived without integrating the variability of both internal and external factors.

## Supporting information

S1 Supporting Information Data sources, detailed methods and additional validations, and supplementary figures and tables. (PDF)
