# Supplementary material for: Telling ecological networks apart by their structure: An environment-dependent approach
Source: PLoS Comput Biol. 2020 Apr 23;16(4):e1007787. doi: 10.1371/journal.pcbi.1007787 (PMC7200011; doi:10.1371/journal.pcbi.1007787)
Supplement: S1 Supporting Information — (PDF) [file pcbi.1007787.s001.pdf]

Supporting Information for

Telling ecological networks apart by their structure: an environment-dependent approach

Contents

S1 Information of empirical networks **S1**

S2 Computation of network metrics **S3**

S3 Correlations among environmental variables **S4**

S4 Separability and scalability using other environmental variables **S7**

S5 Additional analysis on specificity **S10**

S6 Scaling in PCA **S12**

S7 Regression analysis on empirical networks **S14**

S7.1 Regression table S14

S7.2 Regression Model Diagnostics S15

S8 Robustness check of regression analysis on empirical networks **S21**

S8.1 Structural stability of feasibility S21

S8.2 Largest eigenvalue S23

S8.3 Second largest eigenvalue S24

S9 Regression analysis on randomized networks **S26**

S9.1 Erdös-Rényi randomization S26

S9.2 Configuration randomization S27

S1 Information of empirical networks

We have extracted the empirical networks from the public repository `web-of-life.es`. Because this repository is actively updated, here we list the identities of the networks we used. Note that we only used the networks of which we could find their associated environmental information.

Table A: Network labels of mutualistic networks

M_AF_002_01	M_PL_017	M_PL_053	M_PL_061_30	M_SD_001
M_AF_002_02	M_PL_018	M_PL_054	M_PL_061_31	M_SD_002
M_AF_002_03	M_PL_019	M_PL_055	M_PL_061_32	M_SD_003
M_AF_002_04	M_PL_020	M_PL_056	M_PL_061_33	M_SD_004
M_AF_002_05	M_PL_021	M_PL_057	M_PL_061_34	M_SD_005
M_AF_002_06	M_PL_022	M_PL_058	M_PL_061_35	M_SD_006
M_AF_002_07	M_PL_023	M_PL_059	M_PL_061_36	M_SD_007
M_AF_002_08	M_PL_024	M_PL_061_01	M_PL_061_37	M_SD_008
M_AF_002_09	M_PL_025	M_PL_061_02	M_PL_061_38	M_SD_009
M_AF_002_10	M_PL_026	M_PL_061_03	M_PL_061_39	M_SD_010
M_AF_002_11	M_PL_027	M_PL_061_04	M_PL_061_40	M_SD_011
M_AF_002_12	M_PL_028	M_PL_061_05	M_PL_061_41	M_SD_012
M_AF_002_13	M_PL_029	M_PL_061_06	M_PL_061_42	M_SD_013
M_AF_002_14	M_PL_030	M_PL_061_07	M_PL_061_43	M_SD_014
M_AF_002_15	M_PL_031	M_PL_061_08	M_PL_061_44	M_SD_015
M_AF_002_16	M_PL_032	M_PL_061_09	M_PL_061_45	M_SD_016
M_PA_001	M_PL_033	M_PL_061_10	M_PL_061_46	M_SD_017
M_PA_002	M_PL_034	M_PL_061_11	M_PL_061_47	M_SD_018
M_PA_003	M_PL_035	M_PL_061_12	M_PL_061_48	M_SD_019
M_PA_004	M_PL_036	M_PL_061_13	M_PL_062	M_SD_020
M_PL_001	M_PL_037	M_PL_061_14	M_PL_063	M_SD_021
M_PL_002	M_PL_038	M_PL_061_15	M_PL_064	M_SD_022
M_PL_003	M_PL_039	M_PL_061_16	M_PL_065	M_SD_023
M_PL_004	M_PL_040	M_PL_061_17	M_PL_066	M_SD_025
M_PL_005	M_PL_041	M_PL_061_18	M_PL_067	M_SD_026
M_PL_006	M_PL_042	M_PL_061_19	M_PL_068	M_SD_027
M_PL_007	M_PL_043	M_PL_061_20	M_PL_069_01	M_SD_028
M_PL_008	M_PL_044	M_PL_061_21	M_PL_069_02	M_SD_029
M_PL_009	M_PL_045	M_PL_061_22	M_PL_069_03	M_SD_030
M_PL_010	M_PL_046	M_PL_061_23	M_PL_070	M_SD_031
M_PL_011	M_PL_047	M_PL_061_24	M_PL_071	M_SD_032
M_PL_012	M_PL_048	M_PL_061_25	M_PL_072_01	M_SD_033
M_PL_013	M_PL_049	M_PL_061_26	M_PL_072_02	M_SD_034
M_PL_014	M_PL_050	M_PL_061_27	M_PL_072_03	
M_PL_015	M_PL_051	M_PL_061_28	M_PL_072_04	
M_PL_016	M_PL_052	M_PL_061_29	M_PL_072_05	

Table B: Network labels of antagonistic networks

A_HP_001	A_HP_016	A_HP_031	A_HP_046	FW_007
A_HP_002	A_HP_017	A_HP_032	A_HP_047	FW_009
A_HP_003	A_HP_018	A_HP_033	A_HP_048	FW_010
A_HP_004	A_HP_019	A_HP_034	A_HP_049	FW_011
A_HP_005	A_HP_020	A_HP_035	A_HP_050	FW_012_01
A_HP_006	A_HP_021	A_HP_036	A_HP_051	FW_012_02
A_HP_007	A_HP_022	A_HP_037	A_PH_004	FW_013_01
A_HP_008	A_HP_023	A_HP_038	A_PH_005	FW_013_02
A_HP_009	A_HP_024	A_HP_039	A_PH_006	FW_013_03
A_HP_010	A_HP_025	A_HP_040	A_PH_007	FW_013_04
A_HP_011	A_HP_026	A_HP_041	FW_001	FW_013_05
A_HP_012	A_HP_027	A_HP_042	FW_002	FW_014_01
A_HP_013	A_HP_028	A_HP_043	FW_003	FW_014_02
A_HP_014	A_HP_029	A_HP_044	FW_004	FW_014_03
A_HP_015	A_HP_030	A_HP_045	FW_005	FW_014_04

S2 Computation of network metrics

We have used three network metrics in the main text: the largest eigenvalue λ_1, the second largest eigenvalue λ_2, and the structural stability of the intra-guild competition. Note that we only need the binary network to compute these metrics.

To compute the eigenvalues associated with the bipartite networks B, we follow the methods detailed in Supplementary Information S3 in Michalska-Smith and Allesina [12]. Here we briefly Specifically, a bipartite network A can be represented in its matrix form, and then compute the eigenvalues from its associated Laplacian matrix $L := D - A$, where D is the diagonal matrix.

To compute the structural stability of intra-guild competition, we translate the bipartite network into the intra-guild competition matrix. Here the intra-guild competition refers how species in the same guild compete for resources. For example, competition among consumers in antagonistic communities, or competition among pollinators in mutualistic communities. The competition strength is computed, following a niche framework [75], as the relative number of shared resources between two species [55, 56]. Then the structural stability is estimated from the intra-guild competition matrix [76, 77].

S3 Correlations among environmental variables

WorldClim provides 19 environmental variables [33]. These variables are labelled from bio1 to bio19 (see `http://www.worldclim.org/bioclim`). In particular, temperature variability is labelled as bio4. Here we compute the correlations among these variables for the empirical ecological networks. Figures A and B show that many environmental variables are strongly correlated. Figure C shows the correlations among the four environmental variables and the latitude.

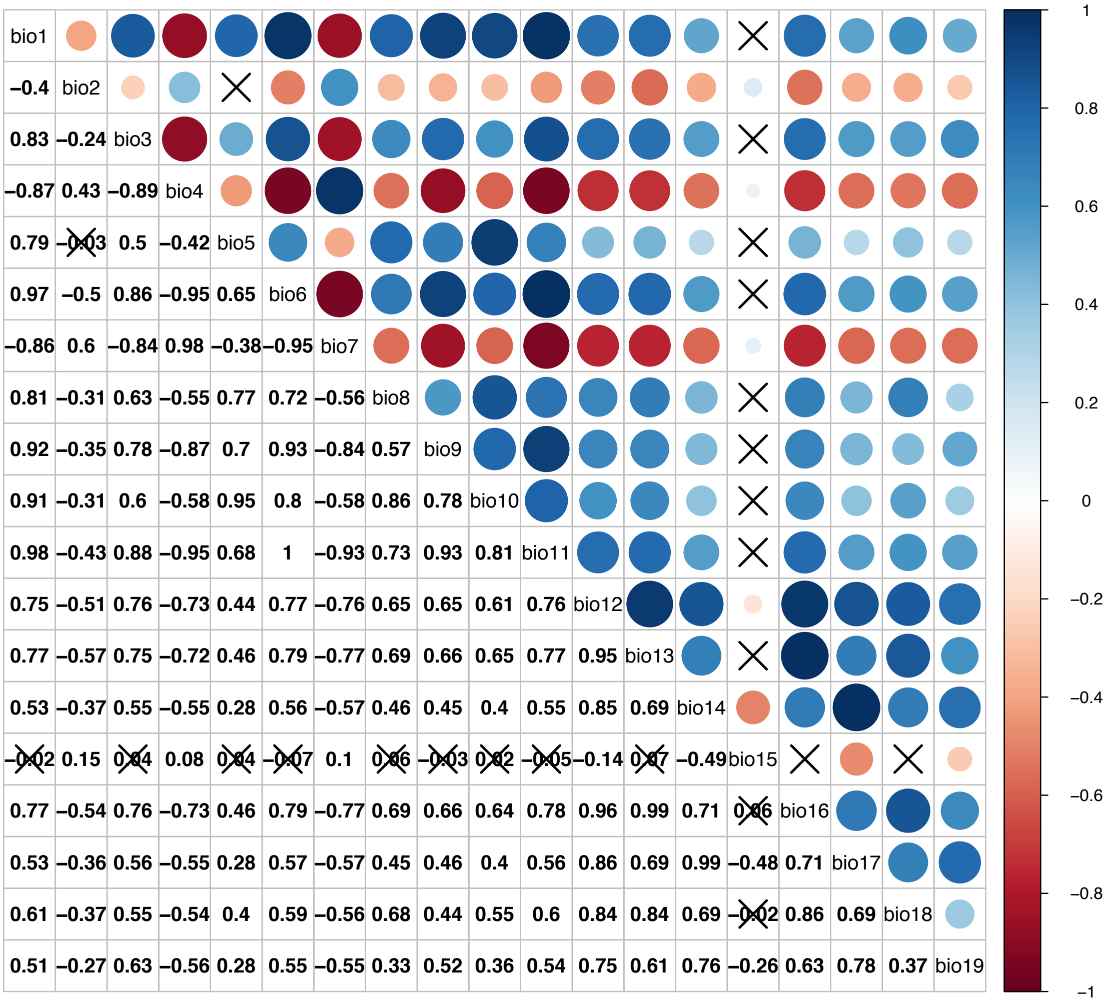

Figure A: **Correlations among environmental variables**. The color of the upper-diagonal element and the numerical value of the lower-diagonal element show the correlation between two environmental variables. The symbol × corresponds to correlations that are not statistically significant at the 5% confidence level.

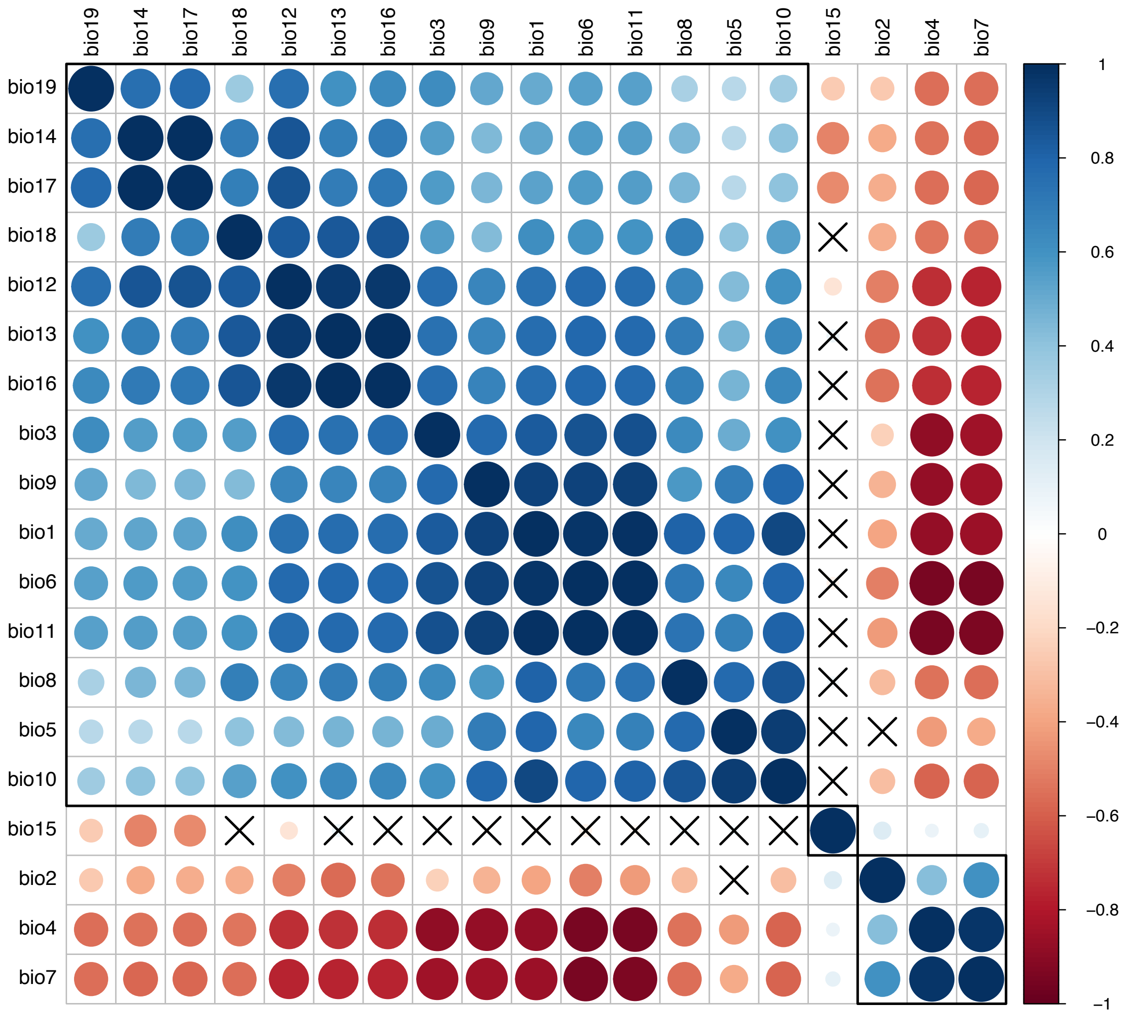

Figure B: **Correlations among environmental variables**. This figure is the same as Figure A except that the environmental variables are arranged into 3 strongly correlated clusters (denoted by the black square).

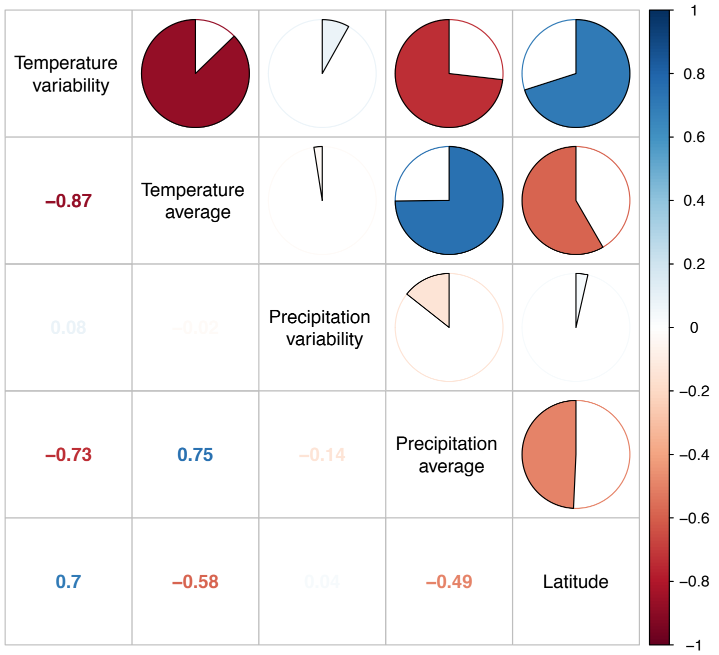

Figure C: Correlation among four environmental variables and the latitude.

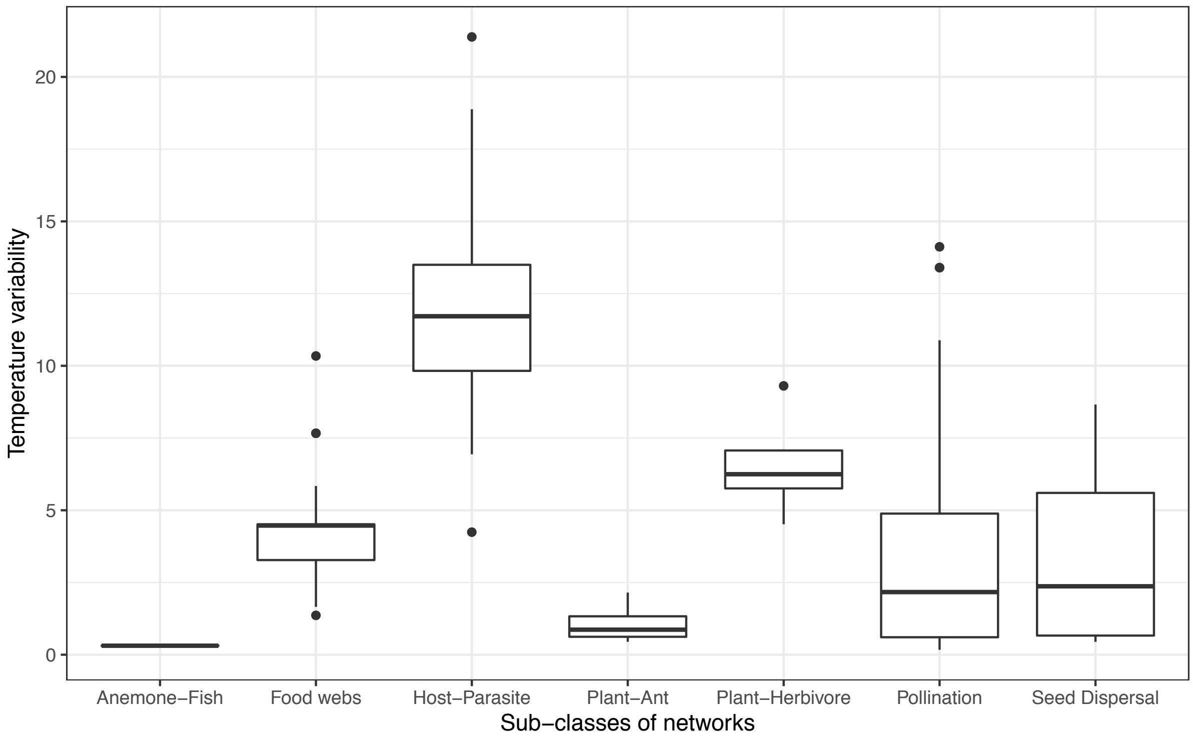

Figure D: The horizontal axis denotes the sub-classes of the networks and the vertical axis denotes the temperature variability where the networks were sampled. The boxplots show the distribution of temperature variability within each sub-class.

S4 Separability and scalability using other environmental variables

Figures E-G show the separability and scalability when other environmental variables are used in the environment-dependent approach. Temperature average (Figure G) and precipitation average (Figure E) work similarly as temperature variability (Figure 3 and 4A). However, precipitation variability (Figure F) does not improve much the separability. Figure C suggests that the poor correlation between precipitation variability and the other environmental variables (temperature average, temperature variability, and precipitation average) may be the reason why.

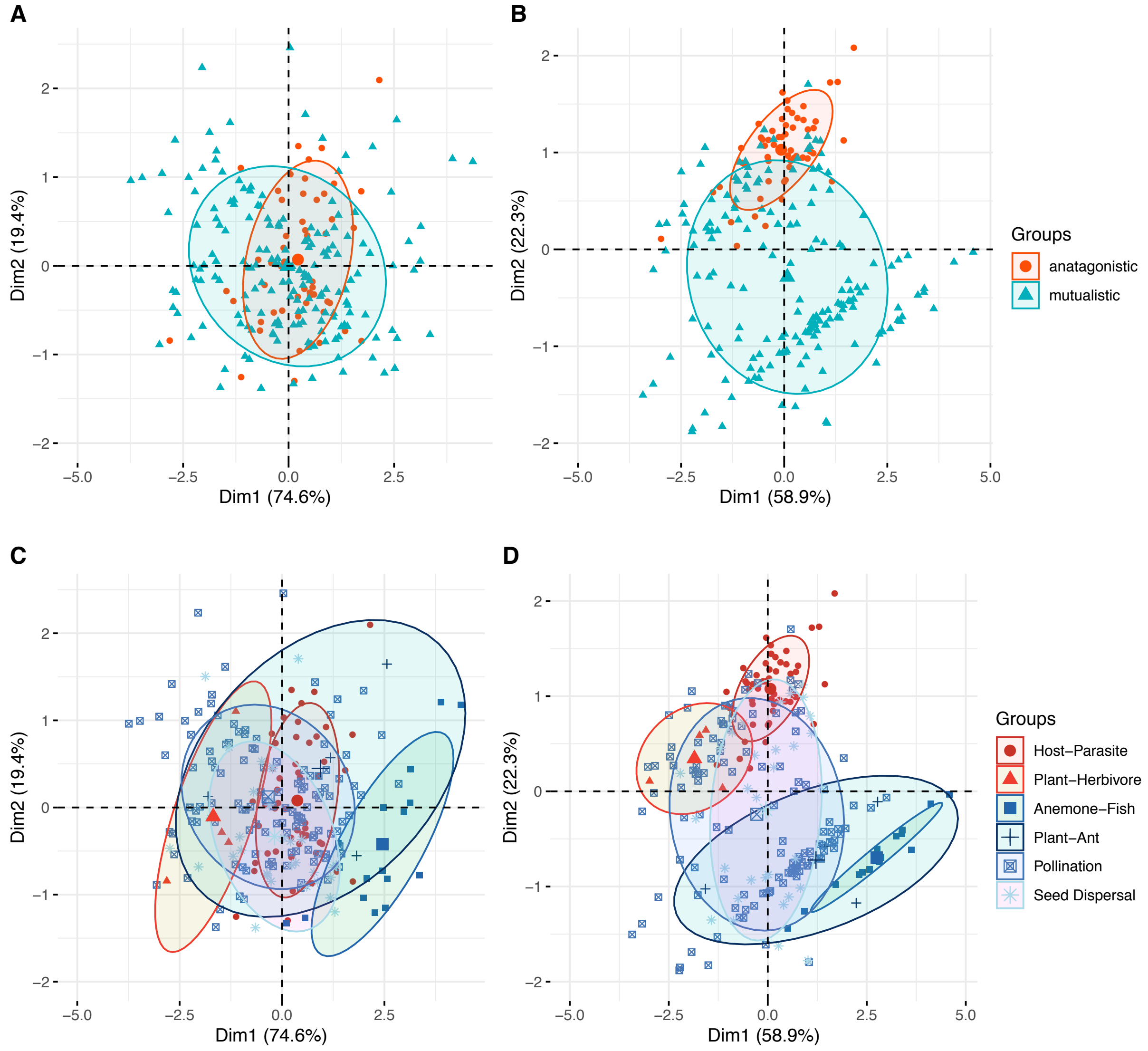

Figure E: Here the environment-dependent approach uses precipitation average as the environmental conditions. Focusing on separability, Panel (A) shows the separability of the environment-independent approach, Panel (B) shows the separability of the environment-dependent approach. Focusing on scalability, Panel (C) shows the scalability of the environment-independent approach, Panel (D) shows the scalability of the environment-dependent approach.

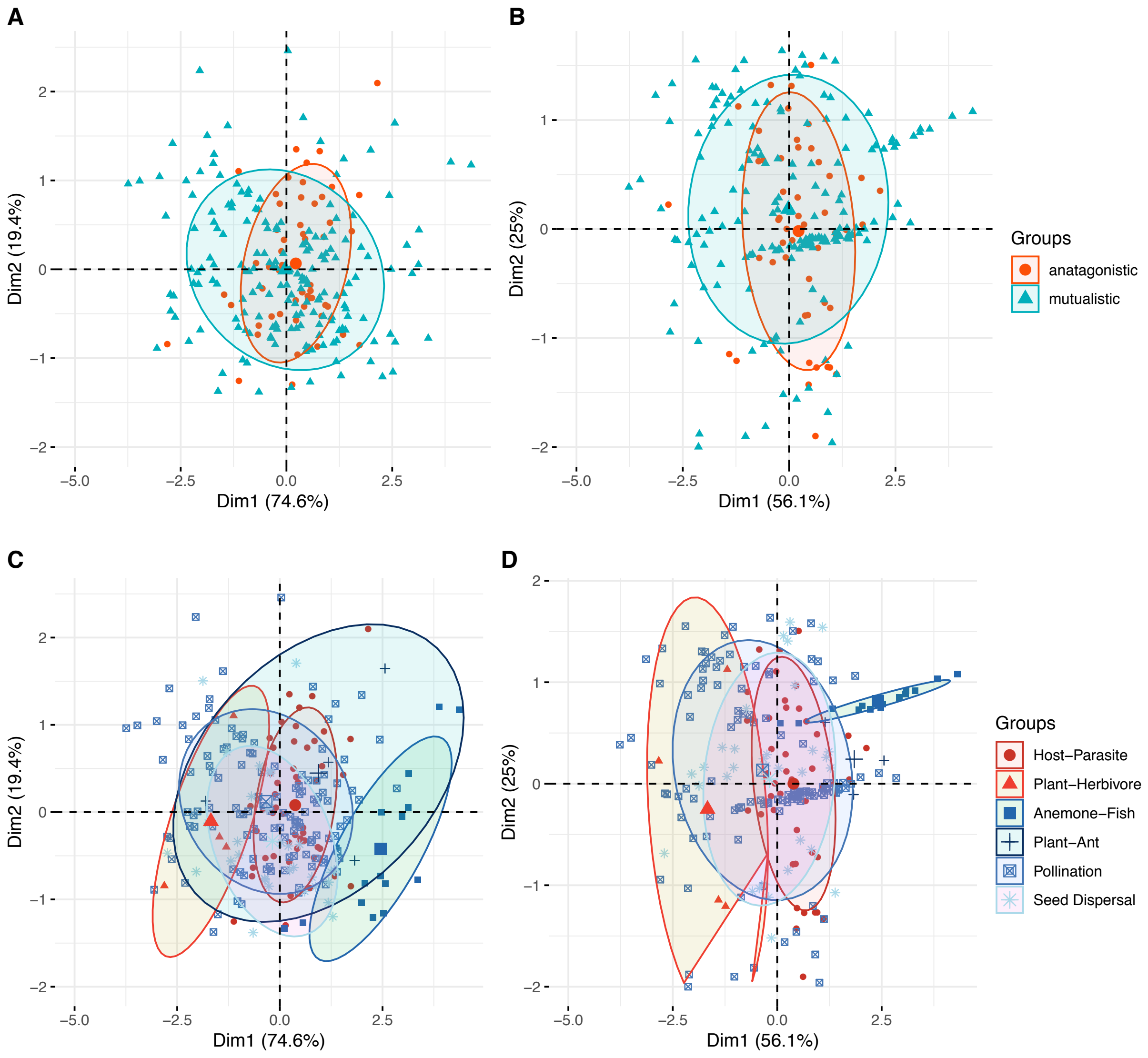

Figure F: Here the environment-dependent approach uses precipitation variability as the environmental conditions. Focusing on separability, Panel (A) shows the separability of the environment-independent approach, Panel (B) shows the separability of the environment-dependent approach. Focusing on scalability, Panel (C) shows the scalability of the environment-independent approach, Panel (D) shows the scalability of the environment-dependent approach.

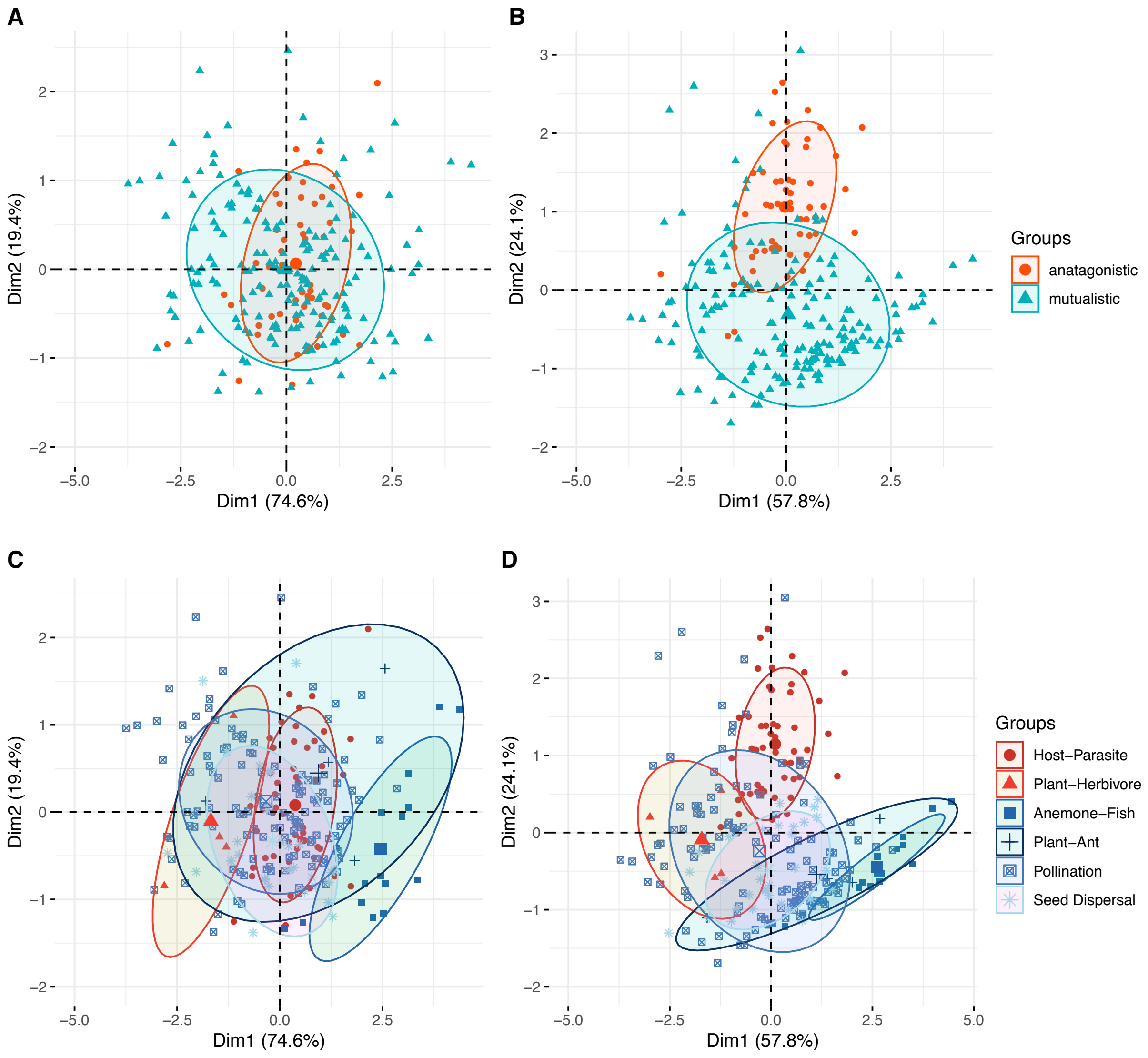

Figure G: Here the environment-dependent approach uses temperature mean as the environmental conditions. Focusing on separability, Panel (A) shows the separability of the environment-independent approach, Panel (B) shows the separability of the environment-dependent approach. Focusing on scalability, Panel (C) shows the scalability of the environment-independent approach, Panel (D) shows the scalability of the environment-dependent approach.

S5 Additional analysis on specificity

Here we are split the networks into a training set (75%) and a test set (25%). We used the Support Vector Machine with a Gaussian kernel. We avoided the data imbalance by keeping the same number of data input from the each community type. To further validate the criterion specificity, we compare four possible cases: (1) randomize the network structure and randomize the temperature variability, (2) randomize the network structure and keep the observed temperature variability, (3) keep the observed network structure and randomize the temperature variability, and (4) keep the observed network structure and keep the observed temperature variability.

Figure H shows how the correct classification percentage changes compare to the baseline. We found that, not surprisingly, "Observed network structure + Observed temperature variability" improves the classification the best and 'Randomized network structure + Randomized temperature variability" improves the classification the worst. We also found that "Randomized network structure + observed temperature variability" improves the classification more than "Observed network structure + Randomized temperature variability"

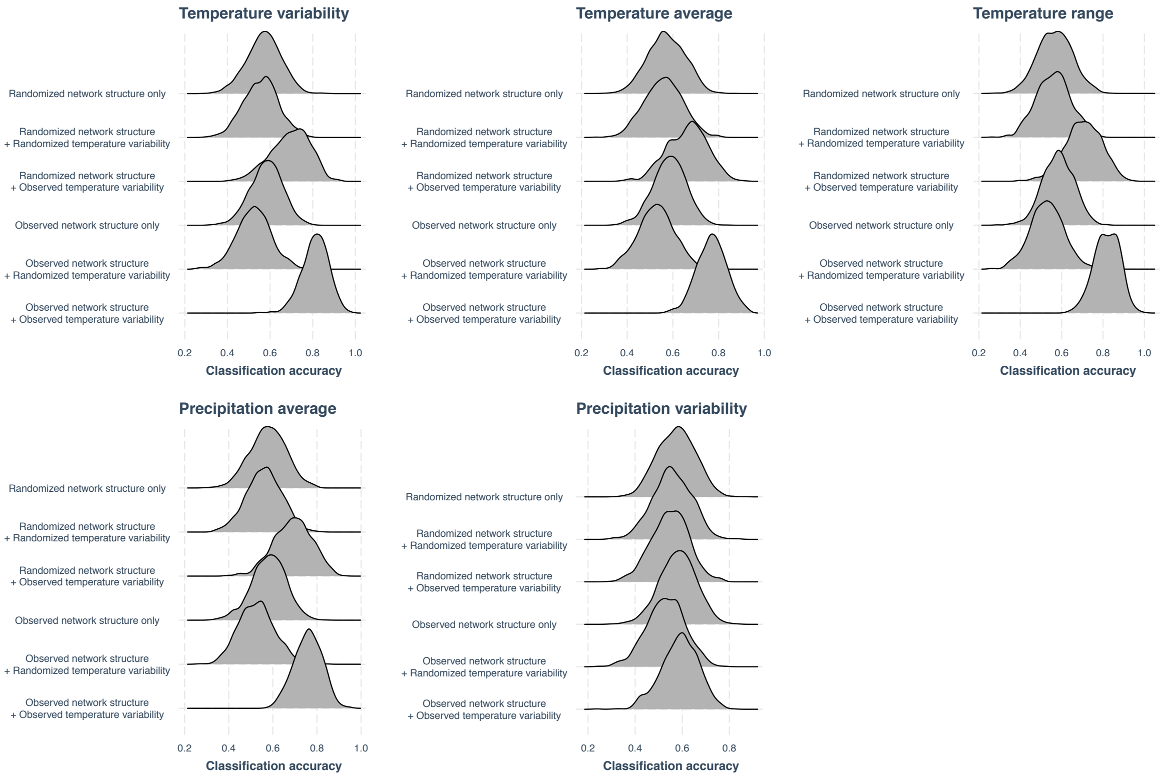

Figure H

We have also tested specificity using the t-Distributed Stochastic Neighbor Embedding (t-SNE) instead of PCA to test the speciality. Qualitative results remain the same.

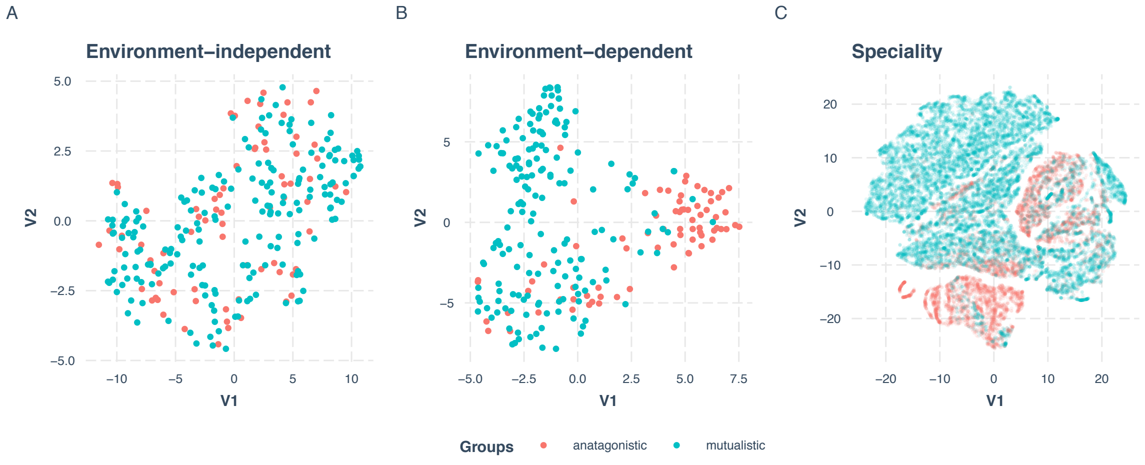
A
Environment–independent
5.0
2.5
0.0
−2.5
−5.0
V2
−10
−5
0
5
10
V1
B
Environment–dependent
5
0
−5
V2
−5.0
−2.5
0.0
2.5
5.0
7.5
V1
C
Speciality
20
10
0
−10
−20
V2
−20
−10
0
10
20
V1
Groups
anatagonistic
mutualistic

Figure I

S6 Scaling in PCA

Here we expand the discussion on the specificity criterion in the environment-dependent approach. To test for specificity, we first randomized the network metrics (by randomizing the network architecture) and kept the environmental information. Then, we used the PCA to differentiate interaction networks. Importantly, the variables should be scaled before performing a PCA [36]. We scaled the variables by their own scaling (linear transformation into mean = 0 and variance = 1). Michalska-Smith and Allesina [12] proposed to scale these variables using the same scaling of the original data. This other scaling is motivated by treating the empirical data as the training set, and randomized networks as the test set [12]. Figure J illustrates the two scaling procedures.

However, these two scaling procedures should not give the same results under the environment-dependent approach. To see why, we need to understand the confounder effects of temperature variability (environmental information) on network class and network metrics (confirmed by the multiple regression). Controlling for this confounder gives us the separability in the environment-dependent approach. Thus, if we randomize the networks and use their own scaling to plot the PCA (the method we used in the manuscript), it is equivalent to making the effect between network metrics and network class weak, while erasing the link between network metrics and temperature variability. This modification makes temperature variability and network metrics independent, limiting the capacity of network metrics to differentiate network class (see Figure JA). But if we use the scaling from the empirical data (the scaling used in Michalska-Smith and Allesina [12]), then we are adding the expectation of network class (i.e., we are conditioning on network class since we have not lost this information). This new scaling (or conditioning) makes temperature variability and network metrics potentially dependent conditional on network class (see Figure JB).

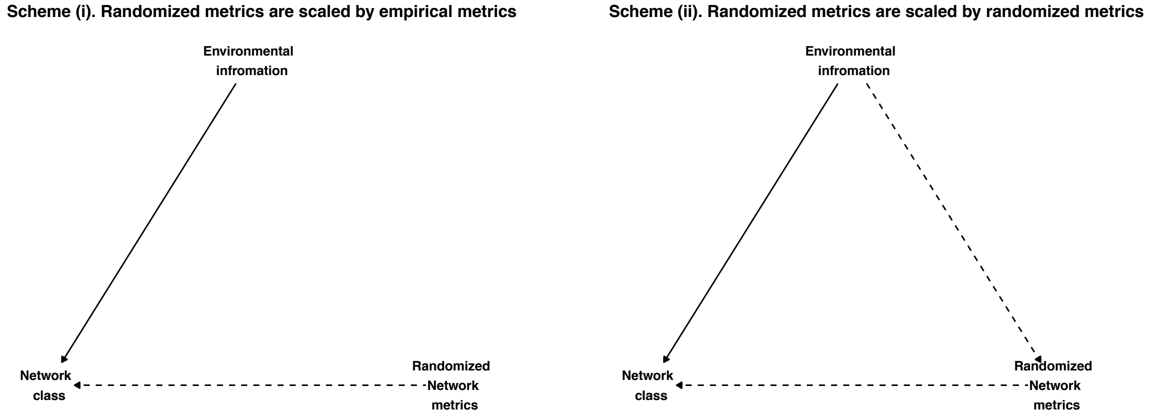

Figure J

Figure K shows that the two scaling procedures give different PCA results. Figure KA reproduced Figure 3B in the main text. Figure KB shows the results when scaled with scheme (i). Figure KC shows the results when scaled with scheme (ii). As discussed above, the circles in KA are considered as the trained models and the randomized networks in KC as treated as the test dataset. Thus, the circles in Figure KC are exactly the same as the ones in KA. The randomized networks in KC that are inside each circles are classified according. Note that while the randomized networks cannot be separated in the two circles in KC, they are well-separated along the second axis (a.k.a Dim2). The reason, as discussed above, is because the scaling in Scheme (ii) causes the potential dependency between temperature variability and network metrics when conditioned on the network classes.

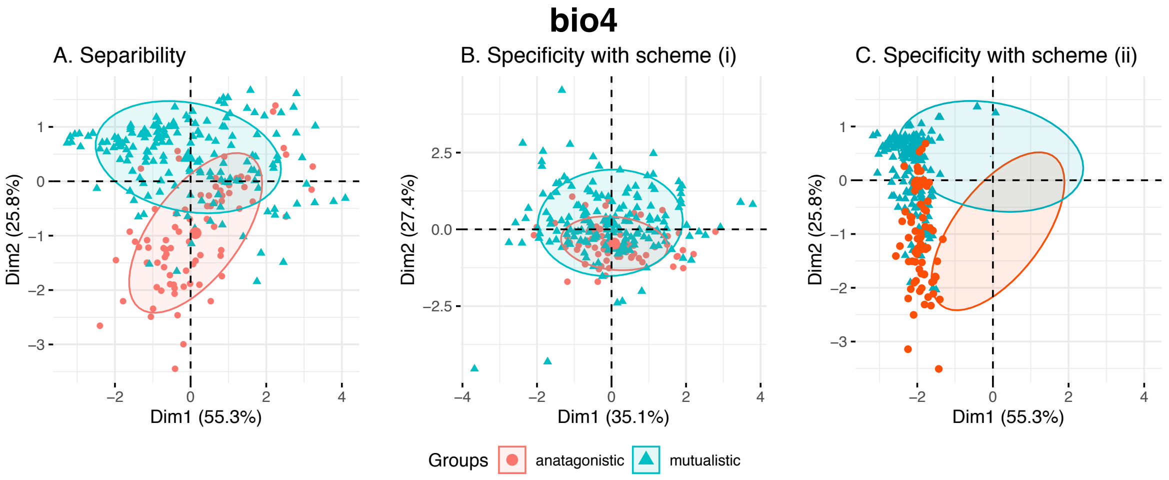

bio4
A. Separibility
B. Specificity with scheme (i)
C. Specificity with scheme (ii)
Dim1 (55.3%)
Dim2 (25.8%)
Dim1 (35.1%)
Dim2 (27.4%)
Dim1 (55.3%)
Dim2 (25.8%)
Groups
anatagonistic
mutualistic

Figure K

S7 Regression analysis on empirical networks

S7.1 Regression table

Table C: **Effect of temperature variability on network stability.** The table summarizes the results of regressing temperature variability on three different metrics of network stability. All variables are scaled. The standard errors of the effects are reported in parentheses, and the $*$ symbol represents the level of statistical significance. For all metrics of network stability (structural stability of feasibility, largest eigenvalue, and second largest eigenvalue), the table shows that increasing temperature variability significantly decreases network stability for mutualistic communities while it increases network stability for antagonistic communities. Regression are performed by including species richness and connectance as independent variables. This table is formatted through `Stargazer` package [78].

	Structural stability of feasibility		Largest eigenvalue		Second largest eigenvalue	
	mutualistic	antagonistic	mutualistic	antagonistic	mutualistic	antagonistic
**Temperature variability**	-0.389^{***}	0.161^{*}	0.268^{***}	-0.177^{***}	0.156^{***}	-0.281^{***}
	(0.127)	(0.083)	(0.055)	(0.063)	(0.053)	(0.051)
Species richness	-0.107	-2.428^{***}	0.161^{***}	3.112^{***}	0.725^{***}	2.422^{***}
	(0.078)	(0.655)	(0.034)	(0.492)	(0.033)	(0.401)
Connectance	0.129	-0.250^{**}	-0.638^{***}	-0.496^{***}	-0.225^{***}	0.038
	(0.085)	(0.115)	(0.037)	(0.087)	(0.036)	(0.071)
Constant	-0.042	-0.862^{***}	-0.056	1.168^{***}	-0.011	0.766^{***}
	(0.096)	(0.142)	(0.042)	(0.107)	(0.040)	(0.087)
Observations	177	75	177	75	177	75
Adjusted R^2	0.124	0.239	0.782	0.820	0.853	0.674
Residual Std. Error	1.018 (df = 173)	0.614 (df = 71)	0.442 (df = 173)	0.461 (df = 71)	0.428 (df = 173)	0.376 (df = 71)

Note: *p<0.1; **p<0.05; ***p<0.01

S7.2 Regression Model Diagnostics

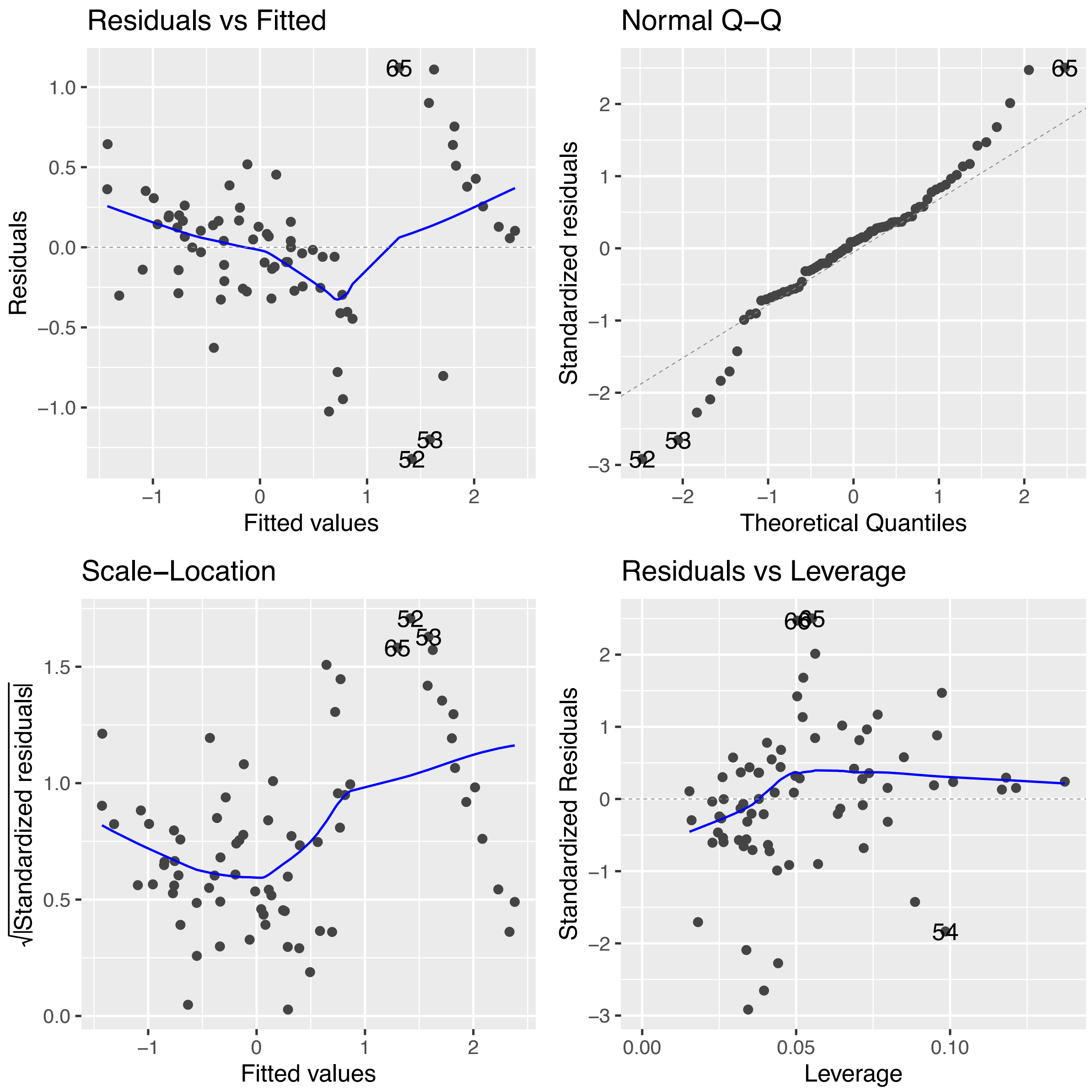

Figure L: Here we check the assumptions of the linear regression when the largest eigenvalue (λ_1) is the dependent variable for antagonistic networks.

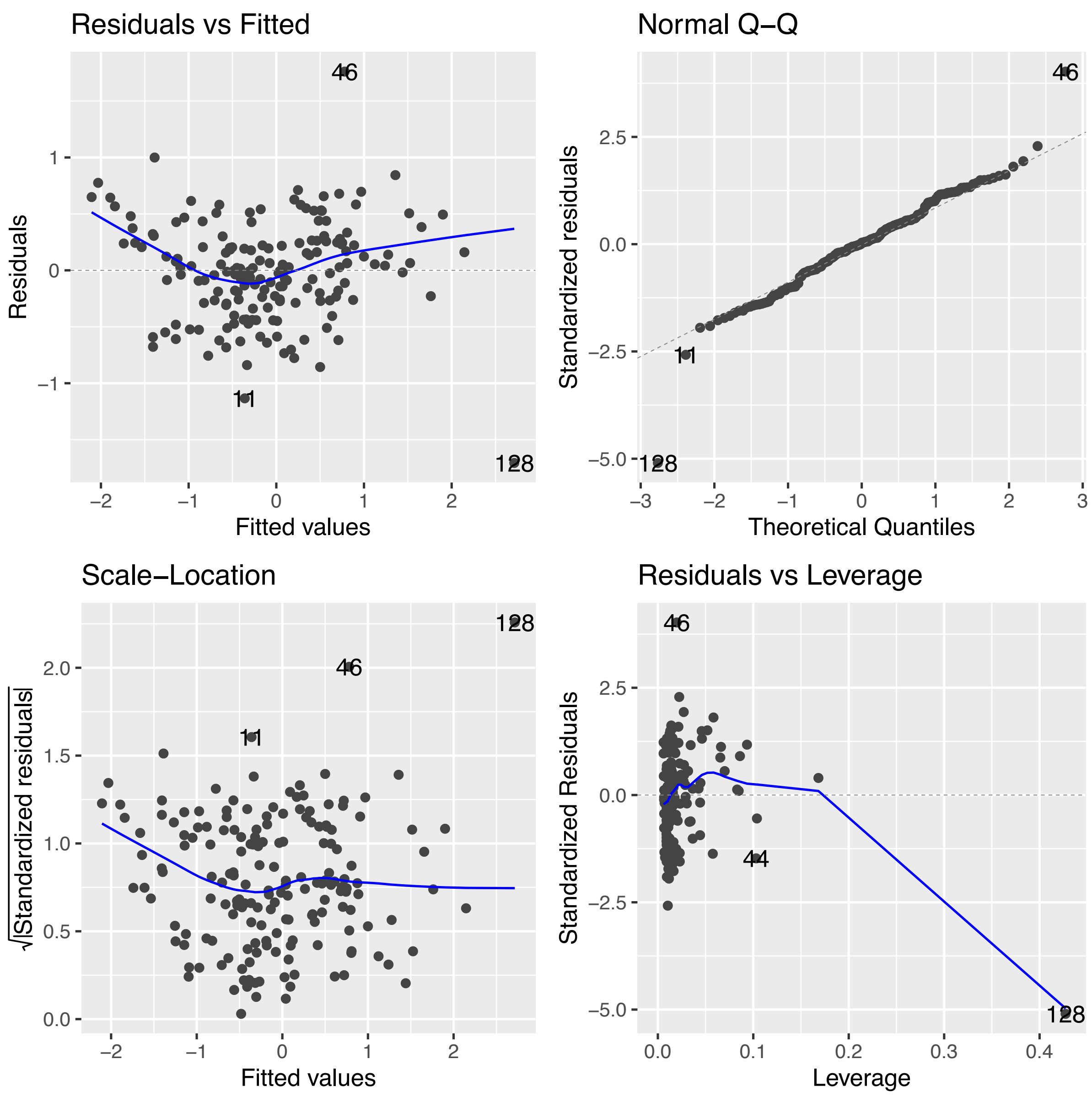

Figure M: Here we check the assumptions of the linear regression when the largest eigenvalue (λ_1) is the dependent variable for mutualistic networks.

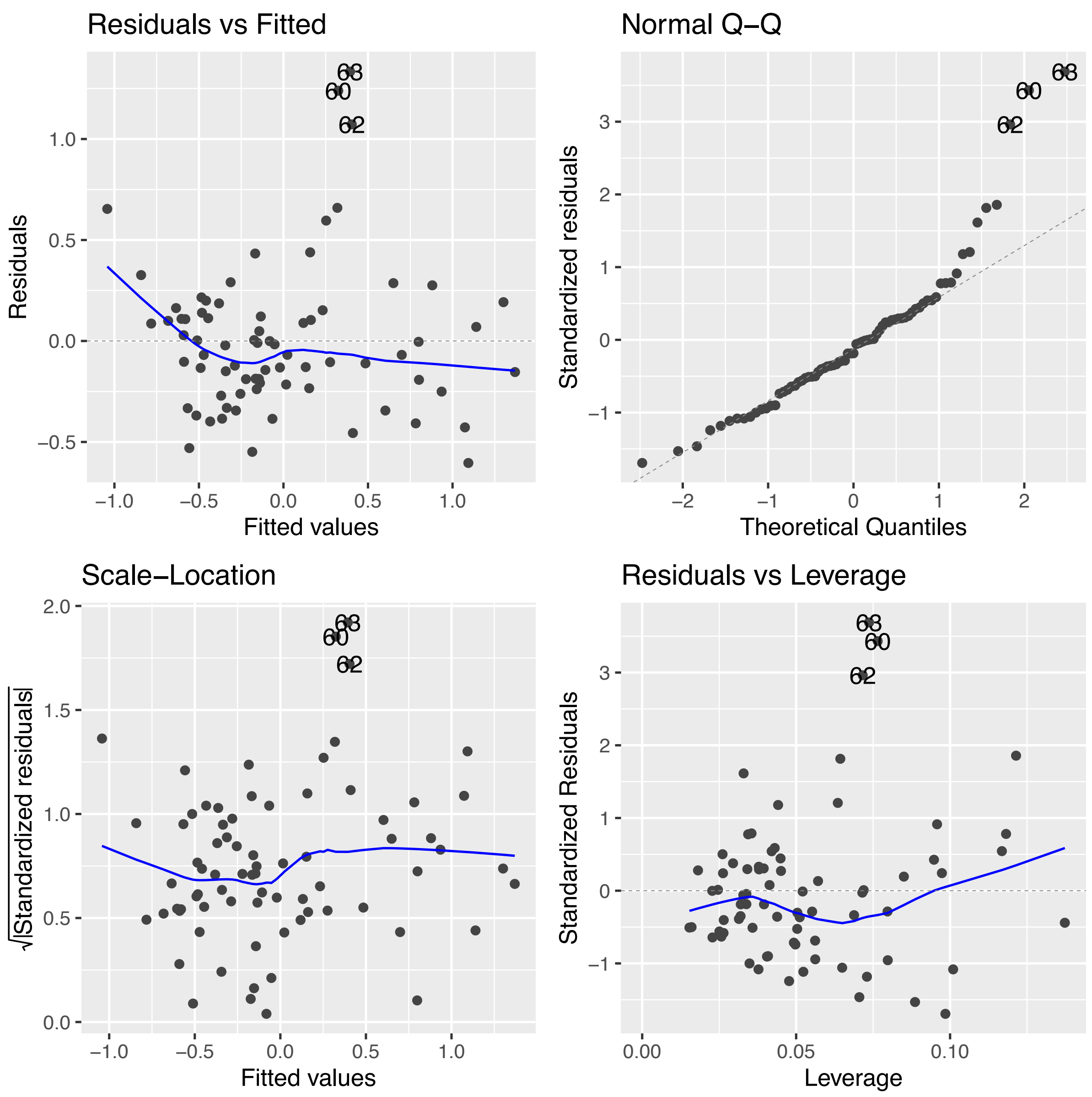

Figure N: Here we check the assumptions of the linear regression when the second largest eigenvalue (λ_2) is the dependent variable for antagonistic networks.

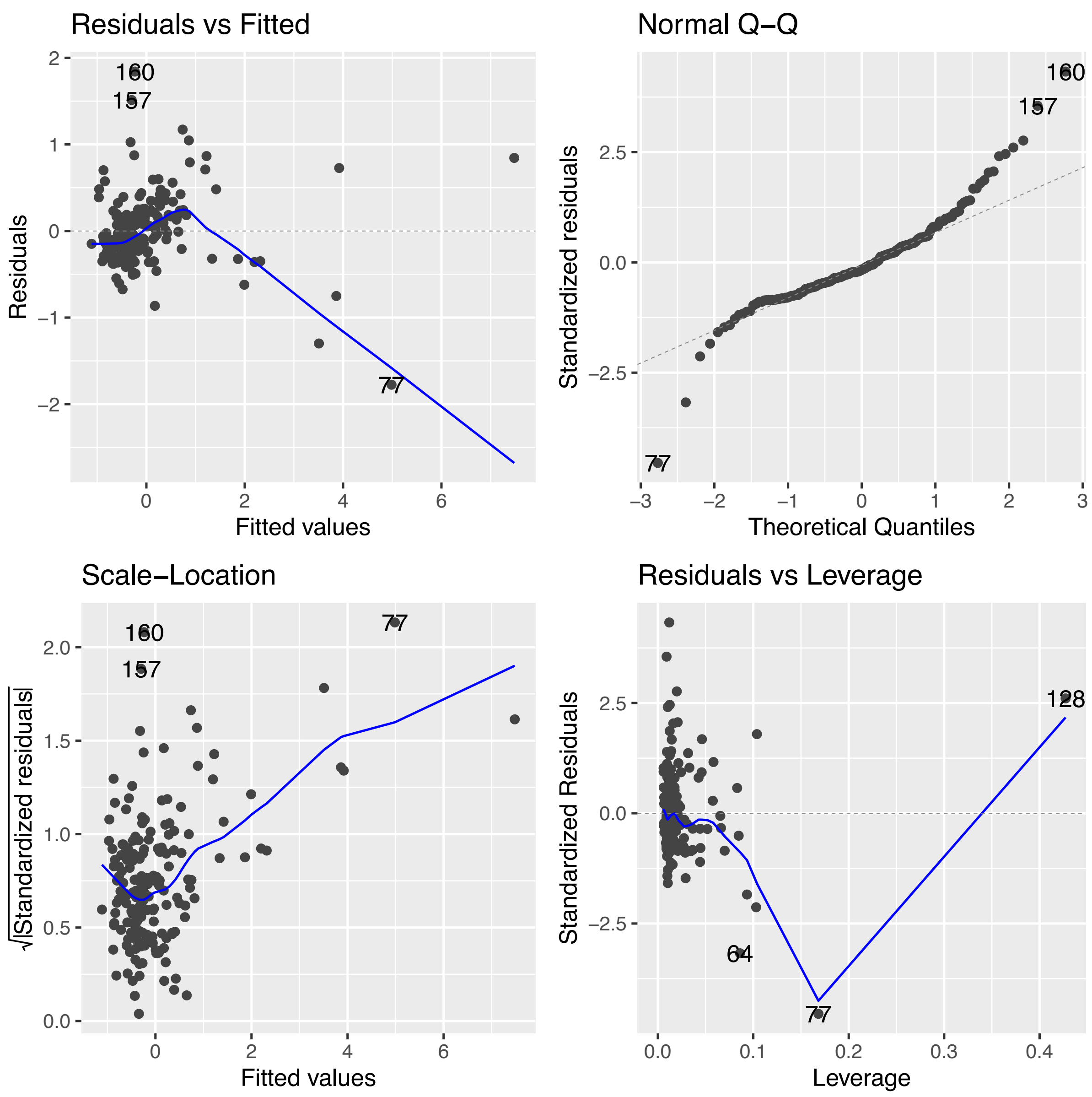

Figure O: Here we check the assumptions of the linear regression when the second largest eigenvalue (λ_2) is the dependent variable for mutualistic networks.

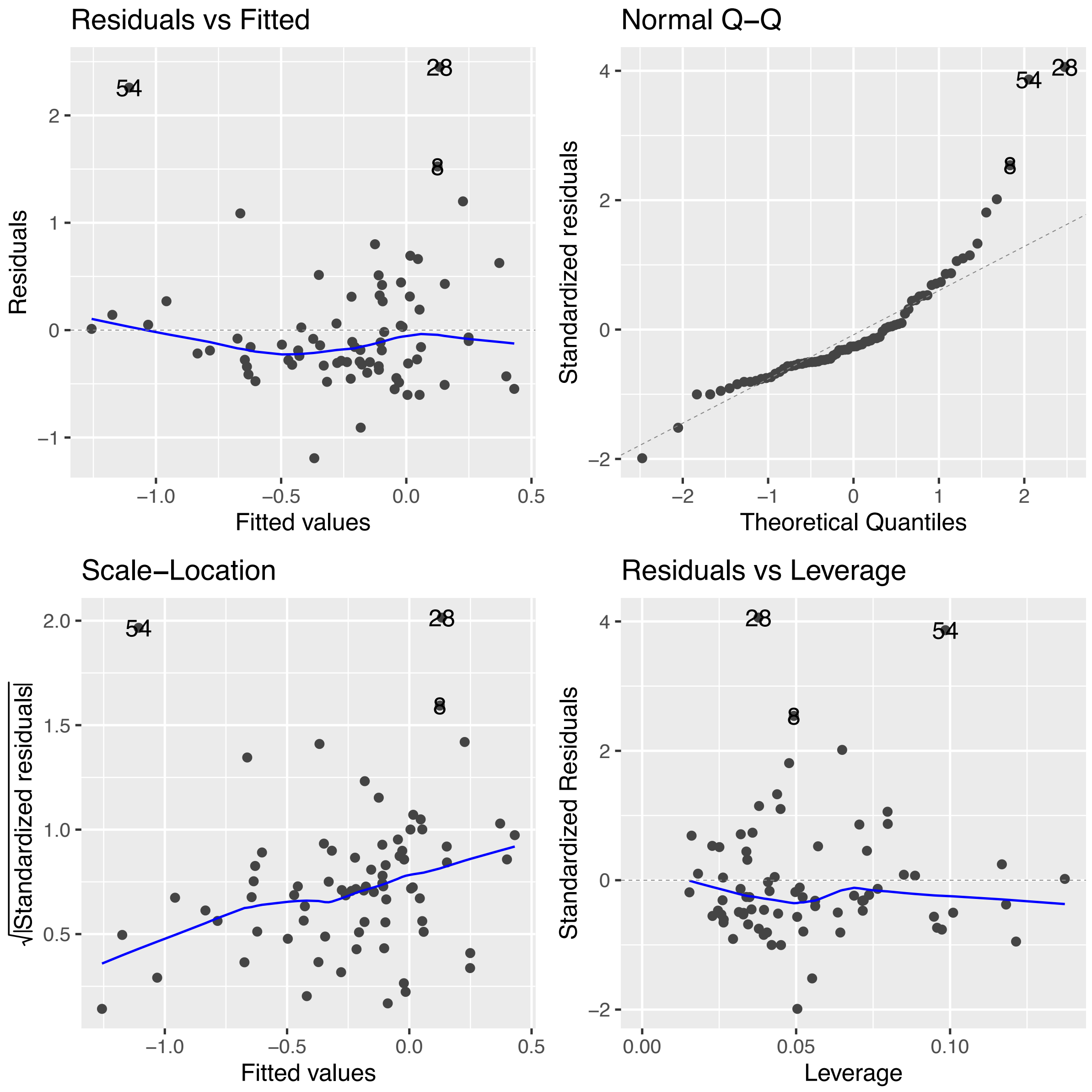

Figure P: Here we check the assumptions of the linear regression when the structural stability (Ω) is the dependent variable for antagonistic networks.

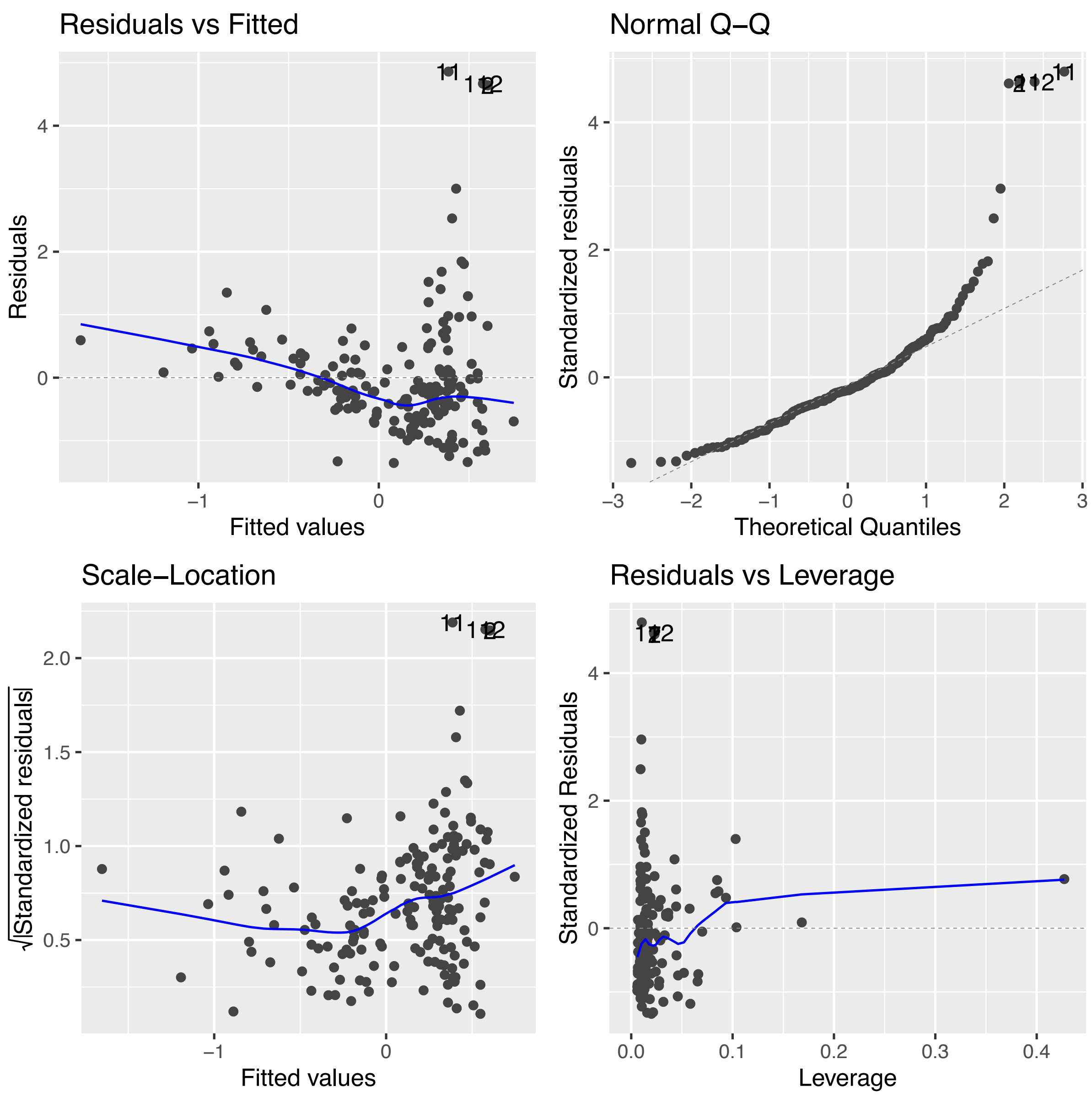

Figure Q: Here we check the assumptions of the linear regression when the structural stability (Ω) is the dependent variable for mutualistic networks.

S8 Robustness check of regression analysis on empirical networks

S8.1 Structural stability of feasibility

Table D: Regression table (similar to Table 1) of structural stability of feasibility on mutualistic networks generated over different models.

	Structural stability of feasibility mutualistic			
Temeprature seasonality	−0.524***	−0.411***	−0.389***	
	(0.116)	(0.126)	(0.127)	
Species richness		−0.154**	−0.107	−0.189**
		(0.072)	(0.078)	(0.075)
Connectance			0.129	0.158*
			(0.085)	(0.086)
Constant	−0.120	−0.058	−0.042	0.133*
	(0.092)	(0.095)	(0.096)	(0.079)
Observations	177	177	177	177
R^2	0.105	0.128	0.139	0.092
Adjusted R^2	0.100	0.118	0.124	0.082
Note:				*p<0.1; **p<0.05; ***p<0.01

Table E: Regression table (similar to Table 1) of structural stability of feasibility on antagonistic networks generated over different models.

	Structural stability of feasibility antagonistic			
Temperature seasonality	0.265***	0.162*	0.161*	
	(0.082)	(0.086)	(0.083)	
Species richness		−1.425***	−2.428***	−2.793***
		(0.475)	(0.655)	(0.639)
Connectance			−0.250**	−0.251**
			(0.115)	(0.118)
Constant	−0.507***	−0.702***	−0.862***	−0.778***
	(0.112)	(0.125)	(0.142)	(0.138)
Observations	75	75	75	75
R^2	0.125	0.222	0.270	0.232
Adjusted R^2	0.113	0.200	0.239	0.211

Note: *p<0.1; **p<0.05; ***p<0.01

S8.2 Largest eigenvalue

Table F: Regression table (similar to Table 1) of largest eigenvalue on mutualistic networks generated over different models.

	Largest eigenvalue mutualistic			
Temperature seasonality	0.662^{***}	0.375^{***}	0.268^{***}	
	(0.094)	(0.090)	(0.055)	
Species richness		0.390^{***}	0.161^{***}	0.217^{***}
		(0.051)	(0.034)	(0.034)
Connectance			-0.638^{***}	-0.658^{***}
			(0.037)	(0.039)
Constant	0.179^{**}	0.023	-0.056	-0.176^{***}
	(0.075)	(0.068)	(0.042)	(0.035)
Observations	177	177	177	177
R^2	0.221	0.414	0.786	0.756
Adjusted R^2	0.216	0.407	0.782	0.754
Note:				$^{*}p<0.1$; $^{**}p<0.05$; $^{***}p<0.01$

Table G: Regression table (similar to Table 1) of largest eigenvalue on antagonistic networks generated over different models.

	Largest eigenvalue antagonistic			
Temperature seasonality	-0.547^{***}	-0.175^{**}	-0.177^{***}	
	(0.120)	(0.075)	(0.063)	
Species richness		5.101^{***}	3.112^{***}	3.514^{***}
		(0.417)	(0.492)	(0.493)
Connectance			-0.496^{***}	-0.495^{***}
			(0.087)	(0.091)
Constant	0.785^{***}	1.484^{***}	1.168^{***}	1.075^{***}
	(0.163)	(0.110)	(0.107)	(0.107)
Observations	75	75	75	75
R^2	0.223	0.747	0.827	0.808
Adjusted R^2	0.212	0.740	0.820	0.802
Note:				$^{*}p<0.1$; $^{**}p<0.05$; $^{***}p<0.01$

S8.3 Second largest eigenvalue

Table H: Regression table (similar to Table 1) of second largest eigenvalue on mutualistic networks generated over different models.

	Second Largest eigenvalue mutualistic			
Temperature seasonality	0.788*** (0.110)	0.194*** (0.059)	0.156*** (0.053)	
Species richness		0.806*** (0.033)	0.725*** (0.033)	0.758*** (0.031)
Connectance			−0.225*** (0.036)	−0.237*** (0.036)
Constant	0.340*** (0.088)	0.017 (0.044)	−0.011 (0.040)	−0.081** (0.033)
Observations	177	177	177	177
R^2	0.225	0.822	0.855	0.848
Adjusted R^2	0.221	0.820	0.853	0.846
Note:				*p<0.1; **p<0.05; ***p<0.01

Table I: Regression table (similar to Table 1) of second largest eigenvalue on antagonistic networks generated over different models.

	Second Largest eigenvalue antagonistic			
Temperature seasonality	−0.446*** (0.064)	−0.281*** (0.051)	−0.281*** (0.051)	
Species richness		2.271*** (0.282)	2.422*** (0.401)	3.060*** (0.455)
Connectance			0.038 (0.071)	0.039 (0.084)
Constant	0.431*** (0.087)	0.742*** (0.074)	0.766*** (0.087)	0.619*** (0.098)
Observations	75	75	75	75
R^2	0.403	0.686	0.687	0.554
Adjusted R^2	0.395	0.677	0.674	0.542
Note:				*p<0.1; **p<0.05; ***p<0.01

S9 Regression analysis on randomized networks

S9.1 Erdös-Rényi randomization

Table J: Regression table on Erdős-Rényi randomized networks

	Structural stability of feasibility		Largest eigenvalue		Second largest eigenvalue	
	mutualistic	antagonistic	mutualistic	antagonistic	mutualistic	antagonistic
Temperature seasonality	-0.200	0.059	-0.053	-0.124	0.310^{***}	-0.101^{*}
	(0.134)	(0.095)	(0.127)	(0.083)	(0.083)	(0.061)
Species richness	-0.026	-1.457^{*}	-0.085	0.034	0.432^{***}	3.791^{***}
	(0.083)	(0.744)	(0.078)	(0.653)	(0.051)	(0.478)
Connectance	0.023	-0.264^{**}	-0.456^{***}	-0.312^{***}	-0.385^{***}	0.272^{***}
	(0.090)	(0.131)	(0.085)	(0.115)	(0.056)	(0.084)
Constant	0.039	-0.594^{***}	0.018	0.026	0.011	0.958^{***}
	(0.101)	(0.162)	(0.096)	(0.142)	(0.063)	(0.104)
Observations	177	75	177	75	177	75
Adjusted R^2	0.005	0.035	0.134	0.215	0.632	0.575

Note: $^{*}p<0.1$; $^{**}p<0.05$; $^{***}p<0.01$

S9.2 Configuration randomization

Table K: Regression table (similar to Table 1) on configuration randomized networks

	Structural stability of feasibility		Largest eigenvalue		Second largest eigenvalue	
	mutualistic	antagonistic	mutualistic	antagonistic	mutualistic	antagonistic
Temperature seasonality	−0.395***	0.099	−0.310**	0.045	0.480***	−0.115
	(0.126)	(0.072)	(0.133)	(0.087)	(0.101)	(0.077)
Species richness	−0.074	−2.081***	0.011	−1.207*	0.144**	3.328***
	(0.078)	(0.562)	(0.082)	(0.680)	(0.062)	(0.602)
Connectance	0.166*	−0.068	0.219**	0.024	−0.401***	0.055
	(0.085)	(0.099)	(0.089)	(0.120)	(0.068)	(0.106)
Constant	0.011	−0.893***	−0.082	−0.379**	0.075	0.995***
	(0.095)	(0.122)	(0.100)	(0.148)	(0.076)	(0.131)
Observations	177	75	177	75	177	75
Adjusted R^2	0.126	0.293	0.077	0.093	0.418	0.501
Note:						*p<0.1; **p<0.05; ***p<0.01
